# Alzheimer's Therapeutics Targeting Amyloid Beta 1–42 Oligomers II: Sigma-2/PGRMC1 Receptors Mediate Abeta 42 Oligomer Binding and Synaptotoxicity

**DOI:** 10.1371/journal.pone.0111899

**Published:** 2014-11-12

**Authors:** Nicholas J. Izzo, Jinbin Xu, Chenbo Zeng, Molly J. Kirk, Kelsie Mozzoni, Colleen Silky, Courtney Rehak, Raymond Yurko, Gary Look, Gilbert Rishton, Hank Safferstein, Carlos Cruchaga, Alison Goate, Michael A. Cahill, Ottavio Arancio, Robert H. Mach, Rolf Craven, Elizabeth Head, Harry LeVine, Tara L. Spires-Jones, Susan M. Catalano

**Affiliations:** 1 Cognition Therapeutics Inc., Pittsburgh, Pennsylvania, United States of America; 2 Mallinckrodt Institute of Radiology, Washington University, St. Louis, Missouri, United States of America; 3 Sanders-Brown Center on Aging, University of Kentucky, Lexington, Kentucky, United States of America; 4 Department of Molecular and Biological Pharmacology, University of Kentucky, Lexington, Kentucky, United States of America; 5 Departments of Neurology and Neuroscience, Massachusetts General Hospital and Harvard Medical School, Boston, Massachusetts, United States of America; 6 Department of Psychiatry, Washington University, St. Louis, Missouri, United States of America; 7 Department of Pathology and Cell Biology and Taub Institute for Research on Alzheimer's Disease and the Aging Brain, Columbia University New York, New York, United States of America; 8 The University of Edinburgh, Center for Cognitive and Neural Systems and Euan MacDonald Centre for Motorneurone Disease, Edinburgh, Scotland; 9 Department of Neurology, Northeastern University, Boston, Massachusetts, United States of America; 10 School of Biomedical Sciences, Charles Sturt University, Wagga Wagga New South Wales, Australia; Huashan Hospital, Fudan University, China

## Abstract

Amyloid beta (Abeta) 1–42 oligomers accumulate in brains of patients with Mild Cognitive Impairment (MCI) and disrupt synaptic plasticity processes that underlie memory formation. Synaptic binding of Abeta oligomers to several putative receptor proteins is reported to inhibit long-term potentiation, affect membrane trafficking and induce reversible spine loss in neurons, leading to impaired cognitive performance and ultimately to anterograde amnesia in the early stages of Alzheimer's disease (AD). We have identified a receptor not previously associated with AD that mediates the binding of Abeta oligomers to neurons, and describe novel therapeutic antagonists of this receptor capable of blocking Abeta toxic effects on synapses *in vitro* and cognitive deficits *in vivo*. Knockdown of sigma-2/PGRMC1 (progesterone receptor membrane component 1) protein expression *in vitro* using siRNA results in a highly correlated reduction in binding of exogenous Abeta oligomers to neurons of more than 90%. Expression of sigma-2/PGRMC1 is upregulated *in vitro* by treatment with Abeta oligomers, and is dysregulated in Alzheimer's disease patients' brain compared to age-matched, normal individuals. Specific, high affinity small molecule receptor antagonists and antibodies raised against specific regions on this receptor can displace synthetic Abeta oligomer binding to synaptic puncta *in vitro* and displace endogenous human AD patient oligomers from brain tissue sections in a dose-dependent manner. These receptor antagonists prevent and reverse the effects of Abeta oligomers on membrane trafficking and synapse loss *in vitro* and cognitive deficits in AD mouse models. These findings suggest sigma-2/PGRMC1 receptors mediate saturable oligomer binding to synaptic puncta on neurons and that brain penetrant, small molecules can displace endogenous and synthetic oligomers and improve cognitive deficits in AD models. We propose that sigma-2/PGRMC1 is a key mediator of the pathological effects of Abeta oligomers in AD and is a tractable target for small molecule disease-modifying therapeutics.

## Introduction

Age-dependent accumulation of amyloid beta 1–42 (Abeta) protein leads to self-association and soluble oligomer formation [1]–[8]. Abeta oligomers bind specifically and saturably to neurons, triggering a variety of changes that result in inhibition of synaptic plasticity [9]–[20] and concomitant failure of memory formation mechanisms in Alzheimer's patients [21]–[26]. Several candidate receptors for oligomers have been proposed in the literature [9],[11],[13],[27]–[29]. In a separate publication we have shown that screening a small molecule library in a phenotypic assay for membrane trafficking in mature (21 days *in vitro*) cultures of neurons resulted in the discovery of distinct classes of novel compounds that exhibit key therapeutic properties: these compounds both prevent and reverse the effects of Abeta oligomers on membrane trafficking, block and displace the binding of Abeta oligomers to neuronal cultures, restore the loss of synapses caused by Abeta oligomers, and restore cognition in mouse models of Alzheimer's Disease [30]. In this study our approach was to use these small molecule drug candidates to identify and characterize the receptors that modulate the binding and neuronal actions of Abeta oligomers [30].

These oligomer blocking molecules bind selectively with high affinity to the sigma-2 receptor. The sigma-2 receptor has been a pharmacological target for treatment of several CNS disorders including anxiety, depression and stroke [31], and was recently identified as the protein PGRMC1 [32]. PGRMC1 is a highly conserved heme-binding protein in the membrane associated progesterone receptor (MAPR) family that has been shown to stabilize surface receptor expression of proteins and directly associates with proteins that regulate membrane trafficking [33],[34]. PGRMC1 is widely expressed in brain at low levels, where it is enriched in the post-synaptic density fraction [35]. It is also expressed outside the CNS, and translocates from the endoplasmic reticulum to the plasma membrane in several cell types [31],[33],[34]. More recently, sigma-2/PGRMC1 has been described as a possible drug target in cancer where it is overexpressed in tumor cells [36], but it has not been previously associated with AD.

We validated this receptor's role in mediating Abeta oligomer-induced signaling by measuring oligomer binding and signaling following siRNA-induced knock-down of PGRMC1 protein, or in the presence of selective antibodies directed against various regions on the PGRMC1 protein. We also examined whether specific PGRMC1 antibodies and sigma-2 selective small molecules were able to displace endogenous human Abeta oligomers from unfixed frozen Alzheimer's patient brain sections. We have identified the sigma-2/PGRMC1 protein as a critical receptor mediating greater than 90% of Abeta oligomer binding to neurons and their downstream synaptotoxic effects. Small molecules that prevent and competitively displace Abeta oligomers from neurons *in vitro* and Alzheimer's patient brain tissue are effective at improving cognitive deficits in animal models of AD following acute and chronic systemic administration [30].

## Materials and Methods

### Animal Welfare

These studies were carried out in strict accordance with the recommendations in the Guide for the Care and Use of Laboratory Animals of the National Institutes of Health, 8^th^ Ed. The protocol was approved by the Institutional Animal Use and Care Committees at Cognition Therapeutics Inc. and the University of Kentucky.

### Neuronal Cultures

All procedures were approved by the Institutional Animal Care and Use and Committee at Cognition Therapeutics and were in compliance with the Office of Laboratory Animal Welfare and the Guide for the Care and Use of Laboratory Animals, Eighth Edition.

Sprague-Dawley rats, 18 days pregnant, were euthanized by CO_2_ asphyxiation followed by cervical dislocation, and embryos were removed. Hippocampus and cortical tissue from the embryo brains were digested in 2.5% Trypsin (Life Technologies) to dissociate cells. Isolated cells were plated at a density of 4.6×10^4^ cells per cm^2^ in 384-well poly-D Lysine coated plates (Greiner) in Neurobasal Media (Life Technologies) supplemented with B27 (Life Technologies), Glutamax (Life Technologies) and antibiotics (penicillin, 50 units/ml and streptomycin 50 µg/ml, Life Technologies). Cultures were maintained at 37°C in 5% CO_2_ with weekly media change for 3 weeks prior to experimentation. These mixed cultures of hippocampal plus cortical neurons and glia were used for all of the *in vitro* experiments described.

### Oligomer Preparation

Abeta oligomers were prepared at high (100 µM) concentration according to published methods [37],[38]. An Abeta monomer film was prepared by evaporating 0.253 mg Abeta 1–42 dissolved in 1,1,1,3,3,3,hexafluoro-2-propanol at room temperature for 20 min using N_2_ gas. The film was then dissolved in anhydrous DMSO and diluted to 100 µM with cold Basal Medium Eagle media (BME, Life Technologies catalog 21010), followed by incubation at 4°C for 24 hour to form oligomers. The resulting oligomer preparations were centrifuged at 16,000×g to pellet any insoluble fibrils and the supernatant was diluted in Neurobasal media prior to addition to cultures at the final concentrations listed in each figure legend. Monomeric Abeta peptides were purchased from California Peptide Inc (catalog number 641-15), American Peptide Company (catalog number 62-0-80), and the University of Pittsburgh Peptide Core facility (primary sequence DAEFRHDSGYEVHHQKLVFFAEDVGSNKGAIIGLMVGGVVIA). All studies using synthetic oligomers were performed with this preparation unless otherwise specified.

### Trafficking Assay

Neurons were treated with compounds and/or Abeta oligomer preparations (0.086% DMSO in culture media) and incubated for 1 to 24 hour at 37°C in 5% CO_2_. Tetrazolium salts (3-(4,5-dimethylthiazol-2yl)-2,5diphenyl tetrazolium bromide, Roche Molecular Biochemicals) were added at a final concentration of 0.75 mM and incubated at 37°C for 60–90 min. Vesicular formazan remaining in cells was quantified by absorbance spectrometry (590 nm with 690 nm subtracted) following extraction with 1.6% Tween-20. All compounds were tested in quadruplicate wells for each concentration in at least 8 replicate experiments with data from all experiments pooled for analysis with means ± S.E.M.

### Sigma-2 radioligand binding

Radioligand competition assays were performed in membranes from Jurkat cells using 5 nM [^3^H]1,3-di(2-tolyl) guanidine in the presence of 1 µM (+)-pentazocine with 10 µM haloperidol to define non-specific binding [39].

### Ex vivo autoradiography studies

Quantitative autoradiography studies on frozen unfixed serial tissue sections from normal (N = 4) or Alzheimer's disease (N = 4) postmortem patients' neocortex were conducted and quantified according to previously published procedures [40]. [^3^H](+) pentazocine was used as the radioligand for sigma-1 receptors and [^125^I]RHM-1 (American Radiolabelled Chemicals, Inc.) or [^3^H]DTG (1,3-Di-(2-tolyl)guanidine (Perkin Elmer) in the presence of 1 µM (+) pentazocine for sigma-2 receptors [32]. Human brain samples for these studies were obtained with written consent as previously published [40].

### Abeta binding Assay

To assess the ability of test compounds to prevent the binding of Abeta oligomers, cultures were treated with compounds for 30 minutes, followed by synthetic Abeta 1–42 oligomer preparation treatment for 60 min (total Abeta concentration 0.5 µM, equivalent to Kd concentration). Alternatively, displacement of prebound oligomers was evaluated by adding oligomers 60 min prior to the addition of compounds, followed by additional 30 minutes incubation. Cells were fixed with 3.75% formaldehyde for 15 min, blocked with 5% normal goat serum and 0.5% Triton X-100 and incubated with primary antibodies for Abeta (1 µg/ml 6E10 or 4G8, Covance catalog numbers SIG-39320 and SIG-39330, respectively), MAP-2 (0.2 µg/ml Chemicon), Synaptophysin-1 (1 µg/ml, Anaspec), glial fibrillary acidic protein (GFAP, 1 µg/ml, Thermo-Fisher) and fluorescently labeled secondary antibodies (2 µg/ml, Invitrogen). Images were acquired on a Cellomics VTi automated microscope with a 20X, 0.75 NA objective and analyzed using ThermoFisher/Cellomics Neuronal Profiling bioapplication set to measure punctate labeling of Abeta and synaptophysin-1 along MAP-2 labeled neurites. For each replicate experiment, at least 100 neurons were sampled from 4 replicate wells for each experimental condition (400 to 500 neurons per experimental condition). The number of replicate experiments is reported for each experiment. All data presented for Abeta binding to neurons represents total intensity of Abeta label in neurite spots per neuron, in relative fluorescent units (RFU), unless otherwise indicated.

For correlation analyses, total intensity of PGRMC1 in cell bodies and in neuronal puncta was calculated for each neuron. Neurons were sorted into bins according to their PGRMC1 expression in each compartment, relative to the expression level in the population of cells, such that bins for −2 SD, −1 SD, −0.5 SD, +0.5 SD, +1 SD, +2 SD and >+2 SD were created. For each binned population, the Mean ± S.E.M. for total PGRMC1 expression in the cell body was graphed vs. the total Abeta labeling intensity in neurite puncta and correlation statistics were calculated.

### siRNA

Neuronal cultures were treated with either a mixture of 4 siRNAs targeted against PGRMC1 (GUCUAGGUCUUGGAUAAUA, GGUUUUACCUCAAAUCAGA, UUAGAAUGCAUGAUGUGUU, CUUCUAUCUGUAGUUAAAA, catalog number A-095365-00-0050) or a non-targeting sequence (UGGUUUACAUGUUUUCCUA, catalog number D-001910-03-50, Accell, ThermoFisher). siRNA were resuspended to 100 µM in RNase free water, shaken for 1.5 hours at 37°C, diluted in media and applied to cells at 1 or 2 uM for 48 hours prior to addition of Abeta oligomers for 30 minutes. Cultures were then fixed and co-immunolabeled with 6E10 antibody to detect Abeta and antibody to detect PGRMC1 as described above and analyzed via automated image processing as described above. For PGRMC1 measurements, total intensity in cell bodies was measured.

### Human tissue *ex-vivo* Abeta competition

Brains from human subjects with a diagnosis of AD (CERAD score “definite” by postmortem neuropathological exam) were obtained through the Massachusetts Alzheimer's Disease Research Center and Massachusetts General Hospital (MGH) Neuropathology Department, and experiments were approved by the Massachusetts General Hospital and Harvard Medical School Institutional Review Board. Serial 10 micron sections of frozen parahippocampal gyrus on glass slides were incubated with identical volumes of PBS containing 5 or 15 µM CT01344, or 1 ng/ml C-terminal antibody to sigma-2/PGRMC1 (Everest Biotechnology EB07207) or vehicle for 60 min at room temperature and then fixed and labeled with an antibody specific to Abeta (AW-7, gift from Dominic Walsh [41]) and Cy3-conjugated secondary antibody. Amyloid plaques were labeled with 0.05% Thioflavin-S (Sigma) in 50% ethanol for 8 min before treatment with 80% ethanol for 30 s. Sections from 6 separate donor brains were used for each treatment group. Fluorescent images of the sections were analyzed in ImageJ [42] analysis software using a custom macro which first identifies dense core plaques labeled by Thio-S and then measures the average Abeta labeling intensity in a 2 micron wide ring surrounding each plaque. A total of 346 to 464 plaques from six donor specimens were analyzed for each treatment group. The median intensities of Abeta labeling in the two micron area surrounding each plaques were calculated for each section (6 sections per treatment group) and subjected to multivariate correlation analysis (Spearman's test) to compare treatment effects across all donor brain samples.

### Statistical Analysis

For all experiments involving quantification of Abeta immunofluorescent intensity, at least four replicate, multiwell plates were analyzed, with a minimum of 4 replicate wells per condition on each plate and 16 fields imaged per well. Averages of total puncta intensity per neuron (approx. 90 neurons per well) were calculated for each well analyzed. These well averages were tested for normality using a KS distance test before being analyzed for treatment differences using ANOVA and Bonferroni's multiple comparison post-test or pairwise Student's t-test as indicated.

## Results

### CogRx compounds are ligands for the sigma-2/PGRMC1 receptor

**Figure 1:**
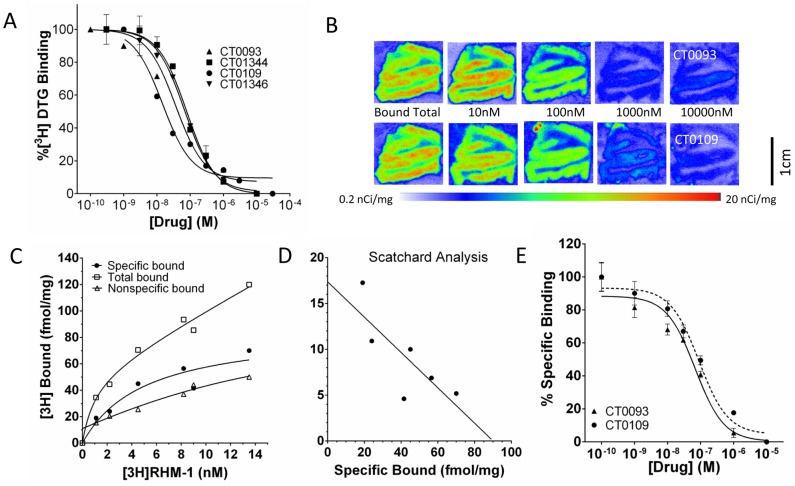
Anti-Abeta compounds are ligands for sigma-2/PGRMC1 receptor. **A**, CT0109, CT0093, CT01344 and CT01346 displace the fiduciary sigma-2 ligand [^3^H]-DTG from receptors on human B cell lines. **B**. Autoradiograms of 18.4 nM [^125^I]RHM-1 binding to human frontal cortex slices in the presence of 10, 100, 1000, 10,000 nM of CT0109 and CT0093, N = 4. Color bar under images show false coloring scale. [^125^I]RHM-1 displays specific saturable binding to human frontal cortex tissue as assessed by quantitative autoradiography in dose-response format (**C**) and as a Scatchard plot (**D**). **E**. Dose response curves for data obtained from autoradiograms in **B**. The Ki's for CT0109 and CT0093 at the [^125^I]RHM-1 binding site were 57±23 nM and 33±12 nM, respectively.

Cognition Therapeutics (CogRx) molecules CT0093, CT0109, CT01344 and CT01346 have been demonstrated to reverse Abeta oligomer-mediated trafficking deficits and restore synapses to normal and restore cognitive function in mouse models of Alzheimer's disease [30]. Active CogRx molecules were counterscreened for activity at 100 central nervous system receptors and enzymes (including those involved in neurotransmission and synaptic plasticity) in industry-standard assays measuring radioligand binding displacement and signaling activity (assays performed at Cerep/Eurofins, Inc., **Table S1**). Active molecules were 10–100-fold selective for the sigma-2/PGRMC1 receptor and competitively displaced selective radioligands from homogenates of a human B cell line (**Fig. 1A**, Ki values (nM): CT0093  = 54±11, CT0109  = 9±7, CT01344  = 48±6, CT01346  = 50±3, mean ± S.E.M.). To determine if these compounds were capable of displacing binding to sigma-2/PGRMC1 receptors in human brain, CT0093 and CT0109 were added in increasing concentrations to serial sections of unfixed frozen cognitively normal elderly human frontal cortex in the presence of a specific radioligand for sigma-2/PGRMC1, ^125^I-RHM-4 [31] (**Fig. 1 **B–D****). CT0109 and CT0093 both displaced the specific binding of this radioligand from human tissue (Ki = 57±23 and 33±12 nM respectively, **Fig. 1B, E**). This evidence demonstrates that these small molecules previously shown to block Abeta oligomer effects *in vitro* and *in vivo* are in fact sigma-2 selective ligands.

### Sigma-2/PGRMC1 is expressed in neuronal cultures and is positively correlated with Abeta oligomer binding

**Figure 2:**
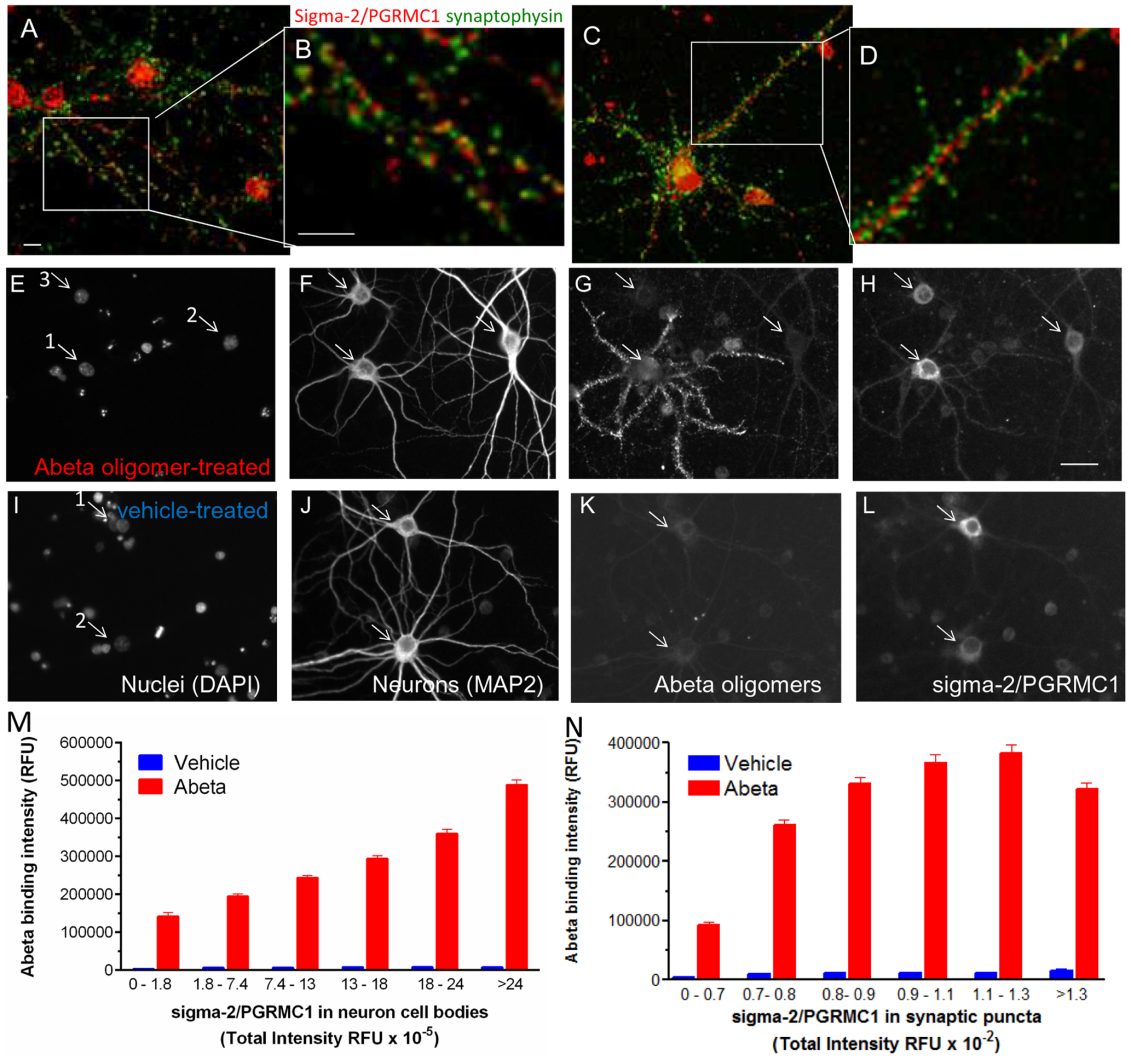
Sigma-2/PGRMC1 protein localizes to synaptic puncta on mature primary hippocampal cultures (21 days *in vitro*) and expression levels are positively correlated with Abeta oligomer binding. sigma-2/PGRMC1 (**A–D**, red) is expressed at low levels in untreated cultures and is localized in cell bodies of neurons and glia, in neurite shafts, and adjacent to presynaptic puncta (**A–D**, synaptophysin  =  green) **B**. 66.7%±2.4 (average ± S.E.M., N = 110 neurons) of PGRMC1 positive puncta on neurons co-localize (yellow) with synaptophysin positive puncta. **E–L**. Positive correlation between sigma-2/PGRMC1 expression and Abeta oligomer binding in neurons (Abeta oligomers  = 400 nM, 1 hour treatment). **E–H**. Only one neuron (MAP2 positive arrow #1 in **E–H**) in this field is labeled with punctate Abeta oligomer binding (**G**), and exhibits elevated PGRMC1 expression (**H**, 3.3×10^5^ RFU) compared to surrounding neurons (#2 = 1.6×10^5^, #3 = 1.8×10^5^ RFU). **I–L**. Vehicle-treated cultures express a similar range of sigma-2/PGRMC1 expression in neurons (arrow #1 in I  = 2.62×10^5^, #2 = 1.21×10^5^ RFU). All scale bars  = 20 microns. **M**, Binning neurons according to their intensity of sigma-2/PGRMC1 immunofluorescence and graphing the average values for Abeta binding from each bin reveals a positive correlation between the intensity of Abeta oligomer binding to synaptic puncta and the expression of sigma-2/PGRMC1 in the cell body that is significant (Kruskal-Wallis, p<0.001). N. A similar analysis of sigma-2/PGRMC1 imunofluorescence in the synaptic puncta also shows a positive correlation with Abeta oligomer binding intensity to synaptic puncta (Kruskal-Wallis p<0.001).

Sigma-2/PGRMC1 has been shown to be expressed in post-synaptic densities in rat brain tissue [35]. Previous reports in tumor cells indicate that PGRMC1 protein is localized to intracellular compartments and the plasma membrane [32]–[34]. Immunofluorescent labeling of untreated 21 days *in vitro* (DIV) mixed hippocampal and cortical cultures with a selective antibody directed against C-terminal amino acids 185–195 showed sigma-2/PGRMC1 is expressed at low levels in cell bodies of neurons and glia, and in proximal neurites (**Fig. 2A–D**, red). In smaller caliber neurites, sigma-2/PGRMC1 can also be detected in immunoreactive puncta adjacent to synaptophysin-immunoreactive (**Fig. 2A–D**, green) presynaptic terminals, consistent with a post-synaptic localization. An average of 66.7%±2.4 (average ± S.E.M., N = 110 neurons) of PGRMC1 positive puncta on neurons colocalize with synaptophysin positive puncta (**Fig. 2A–D**, yellow). Sigma-2/PGRMC1 has a transmembrane region from amino acid 20 to 43 [34], therefore the C-terminus would be predicted to be intracellular, however the orientation of the protein when it is present in the plasma membrane is unclear [34]. Control experiments confirm that a substantial amount of the protein exists at the plasma membrane with the C-terminal end (amino acid 185–195) exposed to the extracellular surface (**Fig. S1**).

Neuronal cultures treated with Abeta oligomers (400 nM) for 1 hour and co-immunolabeled for Abeta, sigma-2/PGRMC1, MAP2 (to distinguish neurons from glia) and DAPI (to label nuclei), exhibit a range of sigma-2/PGRMC1 expression levels in their cell bodies and synaptic puncta (**Fig. 2 E–H**), as do control cultures not treated with Abeta oligomers (**Fig. 2I–L**). We analyzed expression levels of sigma-2/PGRMC1 and binding intensity of Abeta oligomers within each cell to see if they were positively correlated. We focused on the neuronal population for this analysis because 1) oligomers bind specifically and saturably to a single receptor site on neuronal synaptic puncta (Kd = 518±41 nM), whereas the subset of the glial population that binds Abeta does so at 10-fold lower intensities than the binding to neuronal synapses and binds monomer and oligomers equally well, 2) oligomers are 10-fold more potent than monomers at inducing functional changes in membrane trafficking and causing synaptotoxicity and memory deficits *in vivo* [21], 3) oligomer binding to neuronal synaptic puncta is highly correlated with downstream functional changes in membrane trafficking rate, and 4) oligomer binding to synaptic puncta causes synapse regression that is reversible by washout [20],[30]. Examination of individual Abeta oligomer-treated neurons reveals that neurons with more intense Abeta oligomer binding to synaptic puncta had higher expression of sigma-2/PGRMC1 in both their cell body and synaptic puncta (**Fig. 2E–H**). Dividing the cell population into sigma-2/PGRMC1 intensity bins for each compartment (according to the number of standard deviations from the population mean) allows this correlation to be seen quantitatively (**Fig. 2M, N**). This relationship is statistically significant for both the cell body expression of sigma-2/PGRMC-1(Kruskal-Wallis p<0.001) and for expression of sigma-2/PGRMC1 in synaptic puncta (Kruskal-Wallis p<0.001), suggesting that binding of Abeta to hippocampal and cortical neurons is positively correlated with sigma-2/PGRMC1 expression.

### Abeta oligomer treatment causes progressive upregulation of sigma-2/PGRMC1 expression

**Figure 3:**
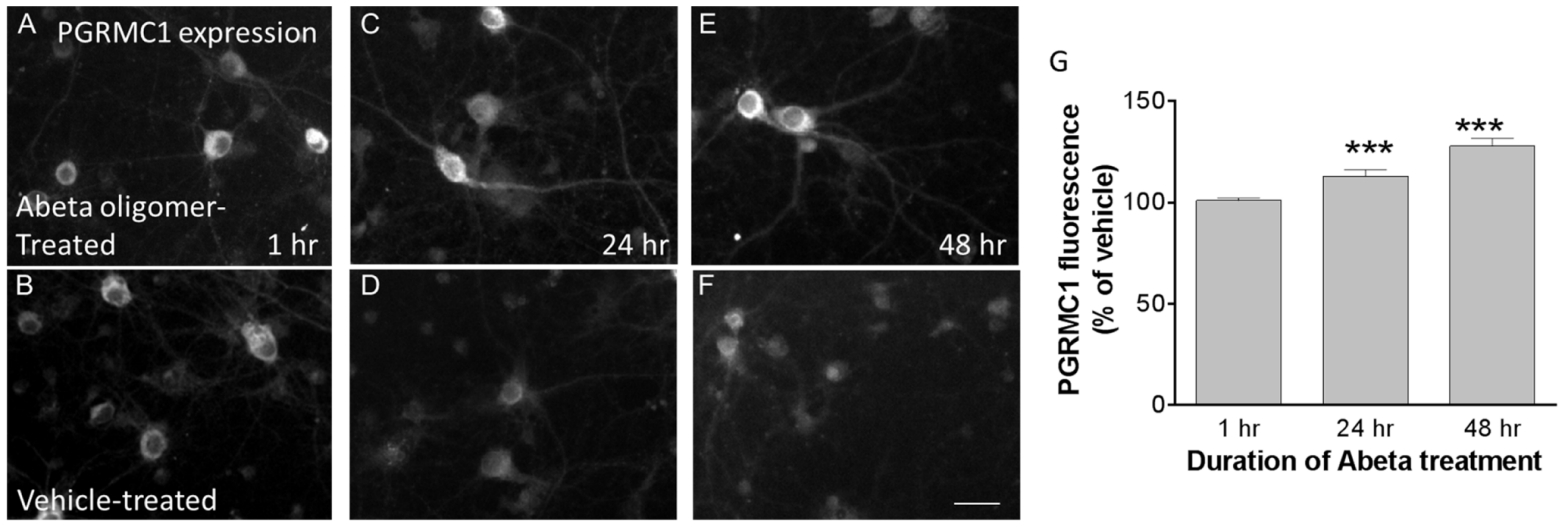
Abeta oligomer treatment causes progressive upregulation of sigma-2/PGRMC1 expression. Expression of sigma-2/PGRMC1 in the cell body of the entire culture cell population significantly increases with time of exposure to Abeta oligomers (**A**, **C**, **E**, 400 nM Abeta oligomers, *p = 0.05, ***p = 0.001, Student's t-test, N>1500 neurons and glia per condition) compared to vehicle-treated cultures (**B**, **D**, **F**). All scale bars  = 20 microns. **G.** Immunofluorescent intensity of sigma-2/PGRMC1 in Abeta oligomer-treated cells, relative to vehicle treatment, at 1 hr, 24 hr and 48 hr.

The total intensity of sigma-2/PGRMC1 expression in the cell body of the pooled population of neurons and glia increases by 13% (±3.2 S.E.M.) after 24 hours of Abeta oligomer exposure and 28% (±3.7 S.E.M.) after 48 hours of exposure, but not after 1 hour (**Fig. 3**). This data suggests that Abeta oligomer treatment causes a progressive upregulation of sigma-2/PGRMC1 expression in neurons.

While Abeta oligomer binding correlates positively with sigma-2/PGRMC1 expression following 1 hour of Abeta oligomer treatment (**Fig. 2**), it is not possible to analyze whether the increase in PGRMC1 expression by treatment with Abeta oligomers at 24 or 48 hours is associated with an increase in Abeta binding. The progressive internalization of bound Abeta oligomers with treatment times greater than 1 hour complicates measurements of binding intensity at these longer time intervals [30].

### Reduction of sigma-2/PGRMC1 protein expression in neuronal cultures decreases binding of Abeta oligomers

**Figure 4:**
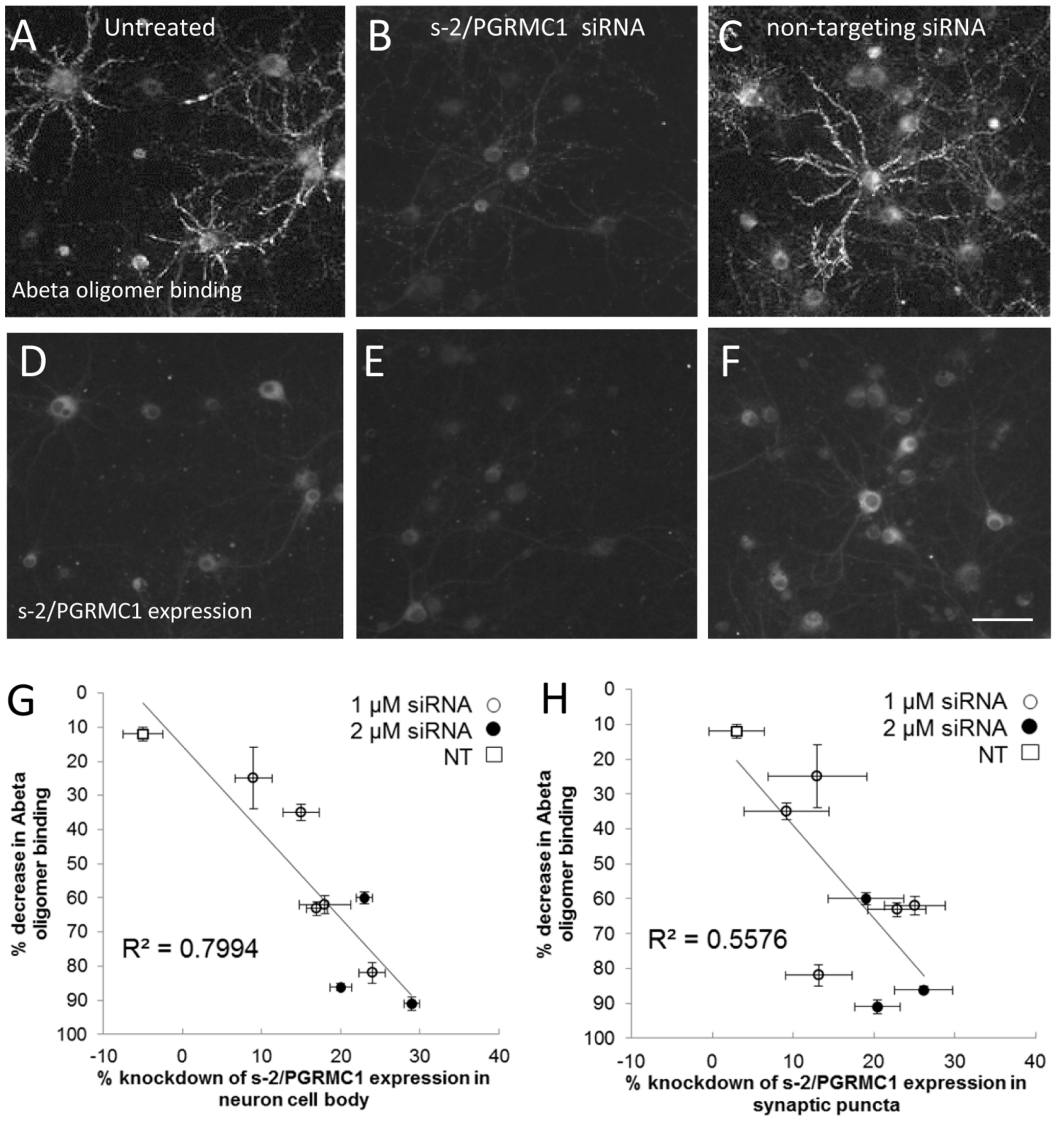
PGRMC1 mediates the binding of Abeta oligomers to neurons *in vitro*. Co-immunolabeling for Abeta oligomer binding (**A–C**) and sigma-2/PGRMC1 expression (**D–F**) in the same field of view in hippocampal and cortical cultures (21DIV). Untreated neurons (**A**, **D**) exhibit Abeta oligomer binding to synaptic sites on neurites and low levels of sigma-2/PGRMC1 expression. In the presence of siRNA to sigma-2/PGRMC1, both Abeta oligomer binding and sigma-2/PGRMC1 expression are significantly reduced (**B, E**). Non-targeting siRNA (**C, F**) has no effect. **G. H**. Graphs of immunocytochemically detectable PGRMC1 protein expression associated with neuron cell bodies (G) and synaptic puncta (H), and Abeta oligomer binding to synapses for each of nine separate experiments (expressed as a percentage of untreated control culture values mean ± S.E.M.). siRNA-mediated reduction in PGRMC1 protein expression of up to 28% results in a corresponding decrease in Abeta oligomer binding by up to 91% (linear regression for PGRMC1 expression in neuronal cell bodies, r^2^ = 0.799, p = 0.0011; for PGRMC1 expression in synaptic puncta, r^2^ = 0.554, p = 0.02).

Since genetic knock-outs of sigma-2/PGRMC1 are not currently available, we examined the role of sigma-2/PGRMC1 protein in mediating Abeta oligomer binding by reducing sigma-2/PGRMC1 protein expression via siRNA-mediated gene silencing in mixed neuronal/glial cultures. Four pooled siRNA sequences to the PGRMC1 protein sequence were added to cultures at 1 or 2 µM final concentration and allowed to incubate for 48 hours to reduce PGRMC1 protein levels via transcriptional suppression. Treated cultures were immunofluorescently labeled to detect Abeta binding and expression of sigma-2/PGRMC1 (**Fig. 4A–F**). Treatment with siRNA resulted in a reduction in immunocytochemically detectable sigma-2/PGRMC1 protein in the neuronal cell body and synaptic puncta of up to 28% (**Fig. 4E**) compared to untreated cultures (**Fig. 4D**), or cultures that had been treated with non-targeting siRNA (**Fig. 4F**). The same cells showed a reduction in Abeta oligomer binding of up to 91% (**Fig. 4B**) compared to untreated cultures (**Fig. 4A**) or non-targeting siRNA treated cultures (**Fig. 4C**). The reduction in sigma-2/PGRMC1 protein expression in neuronal cell bodies and synaptic puncta was highly correlated with the reduction in Abeta oligomer binding (**Fig. 4G**, linear correlation for cell bodies, r^2^ = 0.799, p = 0.0011; **Fig. 4H**, linear correlation for synaptic puncta r^2^ = 0.554, p = 0.02). The lack of a 1∶1 correspondence between loss of PGRMC1 and Abeta oligomer binding could be due to a non-stoichiometric relationship between the two proteins, and/or a differential effect on the brightest Abeta oligomer binding neuronal population, or both (**Fig. S2**). These results demonstrate that Abeta 1–42 oligomers bind directly to sigma-2/PGRMC1 receptors or a protein closely associated with it.

### Sigma-2 receptors are expressed in human brain and are dysregulated in Alzheimer's patients

**Figure 5:**
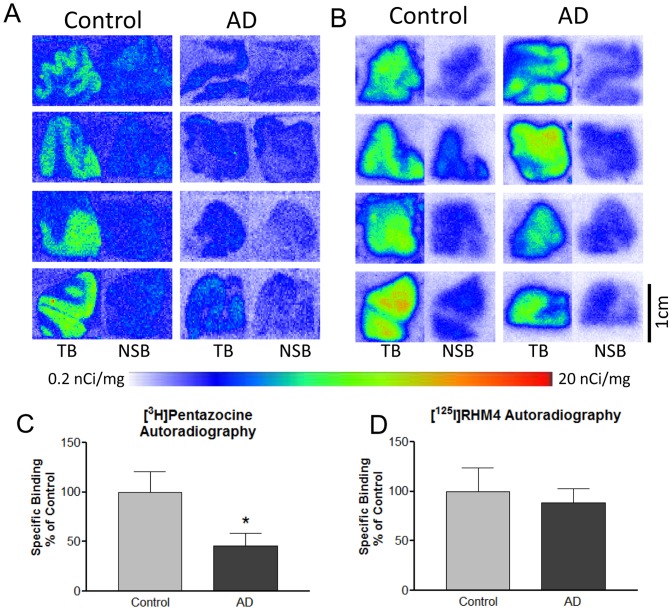
Sigma2/PGRMC1 receptor is present in human neocortex and is upregulated in AD. **A**. Control receptor expression (unrelated receptor sigma-1 labeled with [^3^H]pentazocine 1 nM) declines in advanced AD patient frontal cortex sections vs. age-matched cognitively normal individuals (N = 4, *p = 0.0375, Student's t-test). **B**, adjacent sections from the same individuals labeled with [^125^I] sigma-2 antagonist RHM-4 (0.2 nM) reveal sigma-2 receptor expression is not significantly changed in the disease state vs. normal individuals (N = 4). TB  =  Total binding. NSB  =  non-specific binding in presence of 10 fold excess cold ligand. **C**, **D**, Quantified values for binding in images shown in **A** and **B** expressed as the percent of specific binding in controls.

Serial sections of human frontal cortex from age-matched normal control and AD patient brains were treated with radioligands specific for sigma-2 receptors ([^125^I]RHM-4) or for the unrelated protein sigma-1 ([^3^H]-(+)-pentazocine) and analyzed by autoradiography for binding of the radioligands. Similar to previous reports, sigma-1 receptor exhibited a 54% decline in expression levels in AD patient brains vs. age-matched cognitively normal individuals (**Fig. 5A, C** 4 normal, 4 AD patient brains [CDR stage 3], p = 0.0375, Student's t-test), in parallel to the decline in FDG-PET signal and neuronal loss seen as AD progresses[43]. Because the AD patient brain samples examined are CDR stage 3 (severely demented) and therefore likely have a significant degree of cell loss, this suggests that sigma-1 receptor expression remains constant in surviving neurons in AD brain (however we did not measure cell loss directly in these cases). In contrast, incubation of adjacent tissue sections from these same brains with radiolabeled sigma-2/PGRMC1 ligand [^125^I] RHM-4 [32] (**Fig. 5B, D**) indicates that the level of sigma-2 receptors in AD patients are not different from controls. This could be due to upregulation of sigma-2 receptors in surviving neurons or a downregulation in neurons followed by an upregulation in glia; the present data do not distinguish between these possibilities. This evidence demonstrates that sigma-2 receptors are espressed in human neocortex and may be dysregulated in Alzheimer's disease.

Human mutations in sigma-2/PGRMC1 sequence have not been specifically studied for association with neurodegeneration risk, however there is little tolerated genetic variability in this protein or its family members in the general human population (**Fig. S3**), suggesting that the MAPR proteins (**Table S2**) are essential.

### Abeta oligomer binding to neurons is displaced by sigma-2 selective small molecules and a C-terminal antibody to sigma-2/PGRMC1

**Figure 6:**
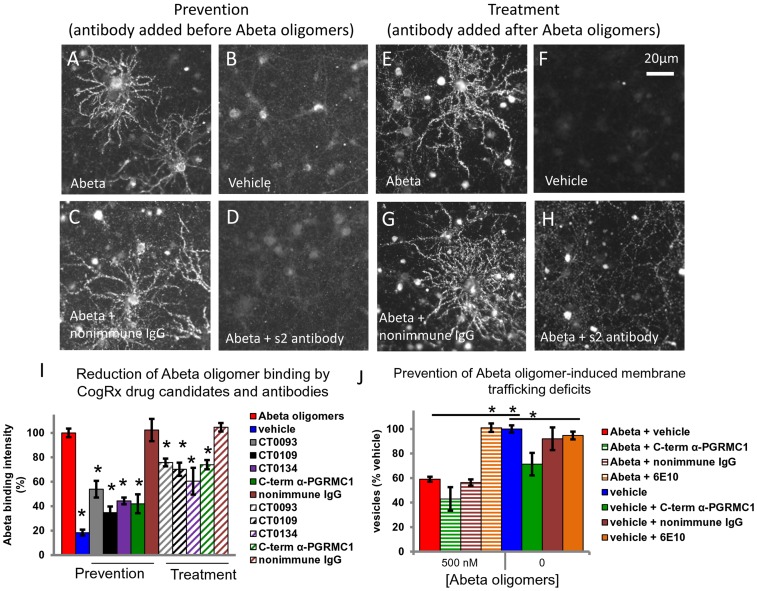
C-terminal antibodies directed against the C-terminus of PGRMC1 prevent (A–D) and displace (E–H) Abeta oligomer binding to neurons and glia. Abeta oligomers bind to a subset of neurons and glia in mature hippocampal primary neurons 21DIV (**A, E, red bar in I**) compared to vehicle-treated (no Abeta) cultures (**B, F**, blue bar in **I**). Graphs in **I** are average of 3 experiments (avg. intensity of Abeta oligomer puncta + S.E.M., expressed as a percentage of Abeta oligomer-treated condition, difference in binding intensity vs. Abeta oligomer condition *p<0.05, Student's t-test). Abeta oligomer binding to cultured neurons is significantly reduced in the presence of C-terminal antibody to sigma-2/PGRMC1 regardless of whether it is added before (**D**, green bar in **I** [prevention], 58% reduction) or after (**H**, green hatched bar in I [treatment], 26% reduction) oligomers. This suggests that oligomers are competitively displaced from receptors at synaptic sites. Non-immune IgG (**C, G** and maroon bars in **I**) and an N-terminal antibody to sigma-2/PGRMC1 (data not shown) cannot reduce oligomer binding under either condition. **J** Effects of antibodies on membrane trafficking rate in the presence or absence of Abeta oligomers (expressed as a percentage of vehicle-treated in the absence of Abeta, difference in trafficking rate vs. Abeta oligomer- or vehicle-treated condition *p<0.05, Student's t-test). The C-terminal antibody directed against amino acids 185–195 in sigma-2/PGRMC1 does not rescue oligomer-induced deficits, but induces trafficking deficits on its own in the absence of Abeta oligomers, pointing to a critical role of this protein in normal membrane trafficking.

We have shown that our sigma-2 selective small molecules are capable of preventing and displacing Abeta oligomer binding to mature primary hippocampal and cortical cultures 21DIV [30]. To confirm the role of the sigma-2/PGRMC1 protein in mediating the binding of Abeta 1-42 oligomers, we treated cultures with Abeta oligomers for 30 minutes, then added a C-terminal specific PGRMC1 antibody to live cultures for 30 minutes, or vice-versa. The cells were then fixed and immunofluorescently labeled to detect Abeta binding (**Fig. 6A–H**). This antibody, which is directed against the C-terminal amino acids 185–195 of sigma-2/PGRMC1, significantly reduced Abeta oligomer binding to synaptic terminals on hippocampal and cortical neurites regardless of whether it was added before (**Fig.** **6D**, green bar in **I** [prevention], 58% reduction) or after (**Fig. 6H**, green hatched bar in I [treatment], 26% reduction) the oligomers. This suggests that oligomers are competitively displaced from receptors at synaptic sites. Non-immune IgG (**Fig. 6C, G** and maroon bars in **I**) and an N-terminal antibody to sigma-2/PGRMC1 (data not shown) cannot reduce oligomer binding under either condition.

We have previously shown that our sigma-2 selective small molecules are capable of preventing and reversing Abeta oligomer-induced membrane trafficking deficits in mature primary 21DIV hippocampal and cortical cultures (a memory and synaptic plasticity-relevant measure), but have no effect on membrane trafficking when added to cultures in the absence of Abeta oligomers [30]. We attempted to test whether blocking Abeta oligomer binding with the C-terminal antibody to PGRMC1 would prevent the downstream functional effects of Abeta oligomers on trafficking. We added the C-terminal antibody directed against amino acids 185–195 of sigma-2/PGRMC1, non-immune IgG or 6E10 antibody (which recognizes amino acids 3–8 on the Abeta sequence) to cultures 1 hour before oligomers, and in the absence of oligomers, and measured the effect on membrane trafficking rate 24 hours later. Abeta oligomers induce a significant deficit in trafficking rate compared to vehicle-treated cultures (**Fig**. **6J**, red bar vs. blue bar). This oligomer-induced trafficking deficit is prevented by preincubation of the cultures with 6E10 (**Fig** **6J**, red bar vs. orange striped bar), but not with C-terminal antibody to sigma-2/PGRMC1, or by nonimmune IgG (**Fig. 6J**, red bar vs. green and maroon striped bars respectively). However, in the absence of Abeta oligomers, the C-terminal antibody to sigma-2/PGRMC1 induces trafficking deficits all on its own (**Fig.** **6J**, blue bar vs. green bar), while nonimmune IgG and 6E10 do not (**Fig** **6J**, blue bar vs. maroon and orange bars). This suggests that the observed induction of trafficking deficits is unlikely to be due to a nonspecific effect of IgG molecules. When added to living cells, the C-terminal antibody to sigma-2/PGRMC1 does not appear to block Abeta-oligomer-induced deficits because like oligomers it accelerates exocytosis rate. Small molecule sigma-2/PGRMC1 ligands do not affect the exocytosis rate in the absence of Abeta oligomers.

These results highlight the critical role that sigma-2/PGRMC1 plays in the synaptic plasticity-relevant process of membrane trafficking. Sigma-2/PGRMC1 protein directly associates with proteins that regulate membrane trafficking [33],[34], translocating from endoplasmic reticulum to the plasma membrane [31],[33],[34] and stabilizing surface receptor expression of proteins. The bivalent binding sites of whole IgG molecule may attach to two PGRMC1 molecules at the plasma membrane extracellular surface and trap the molecule at the surface or induce internalization, or affect the protein's interaction with other proteins, altering the overall process of membrane trafficking. However this does not preclude the possibility that smaller IgG molecules such as Fab fragments could be therapeutically efficacious at blocking Abeta oligomer-induced trafficking deficits.

### Endogenous Abeta oligomers are displaced from human AD patient brain sections by a CogRx compound and antibody to sigma-2/PGRMC1

**Figure 7:**
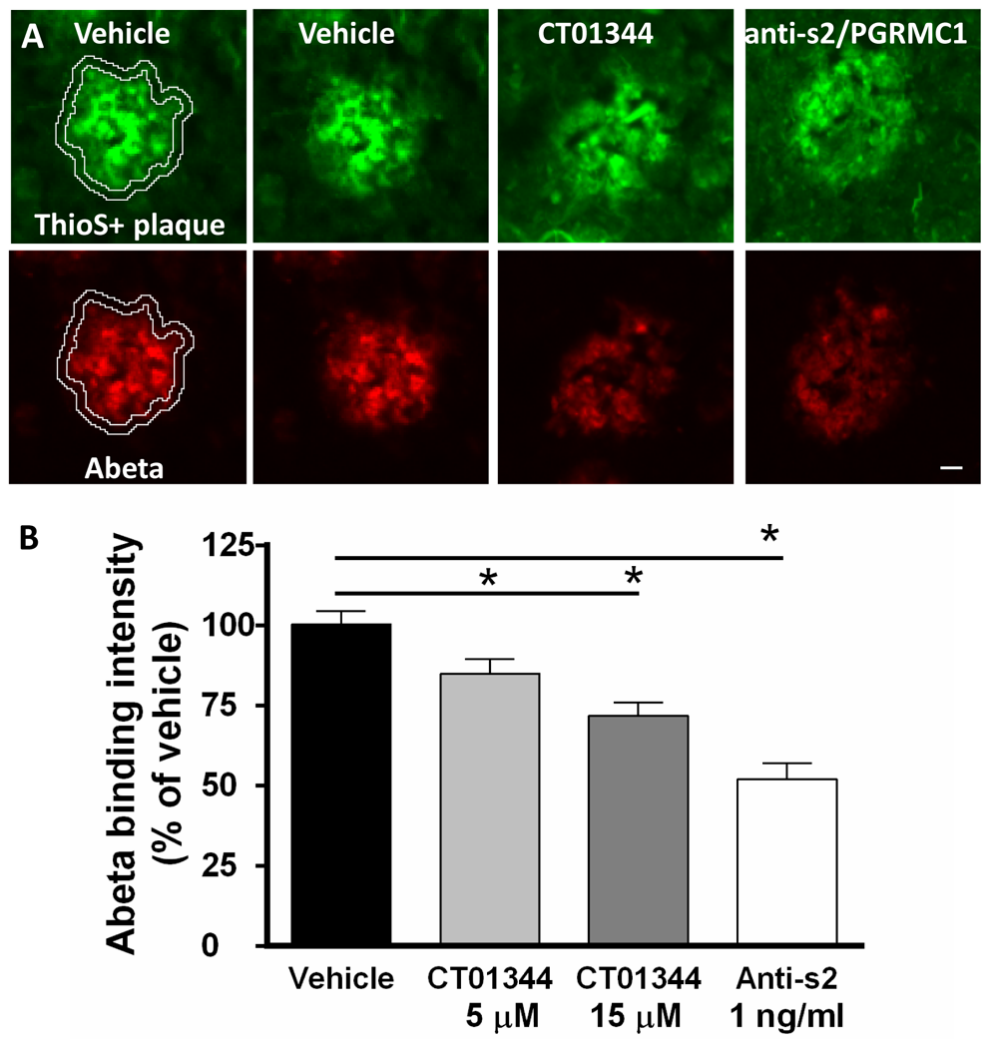
Antibodies and compounds directed against sigma-2/PGRMC1 dose-dependently displace endogenous human Abeta oligomers from specific tissue locations in Alzheimer's brain neocortical tissue sections. **A**, Abeta oligomers located in a 2 micron halo surrounding compact thioflavin-S positive plaques is displaced from frozen postmortem human AD brain tissue sections (N = 7 patients) by C-terminal sigma-2/PGRMC1 antibody and receptor antagonist CT01344 (**B**) in a dose-dependent manner compared to vehicle-treated brain sections from the same individual (Spearman's rank order, rho  = −0.492, p = 0.038). All scale bars  = 20 microns. See **Fig. S4** for details on quantification method.

Koffie et al. [44], reported that a 2 micron wide halo around dense-core thioflavin-S positive plaques in human AD patient brain contain an increased concentration of Abeta oligomers bound to neurons that can be visualized with immunohistochemical detection methods. This same region is associated with a decreased number of synapses [44],[45]. We tested whether CT01344 and the C-terminal antibody to sigma-2/PGRMC1 were capable of displacing endogenous Abeta oligomers from this halo region around amyloid plaques in human AD patient brains (see **Fig S4** for patient characterization and description of analysis algorithm) under non-equilibrium conditions. Adjacent tissue sections of unfixed postmortem neocortex were incubated in a fixed volume of solution containing increased concentrations of CT01344, antibody or vehicle for 1 hour then fixed and immunolabeled for Abeta and co-stained with Thio-S to detect amyloid plaques (**Fig. 7A**). Abeta intensity in the plaque halo region was measured via automated image processing and graphed as a percentage of vehicle values (**Fig. 7B**). CT01344 dose-dependently reduced Abeta immunofluorescence in the 2 micron wide halo (Spearman's Rho  = −0.492, p = 0.038) and the antibody also reduced Abeta immunofluorescence in the halo (p<0.05, Student's t-test). These results demonstrate that endogenous human Abeta oligomers can be dose-dependently displaced from tissue binding sites by sigma-2/PGRMC1-selective small molecules and antibodies directed against the C-terminal region of this protein.

### Behaviorally efficacious sigma-2/PGRMC1 selective small molecules are functional antagonists at the receptor

**Figure 8:**
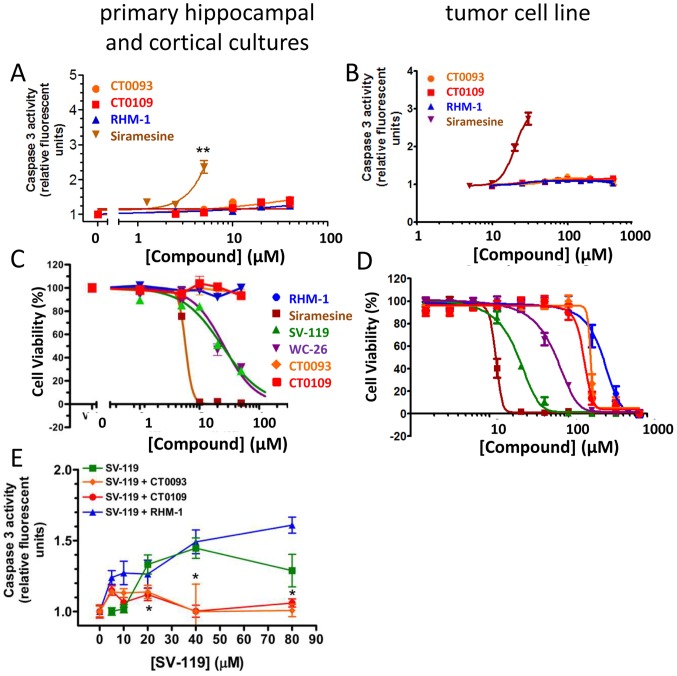
CogRx sigma-2/PGRMC1-selective small molecules are functional antagonists. **A, B** Sigma-2/PGRMC1 agonist siramesine causes dose-dependent activation of caspase 3 in primary neuronal cultures (**A**) and in SKOV-3 human ovarian cancer cells (**B**) but sigma-2/PGRMC1 antagonists RHM-1, CT0109 and CT0093 do not. **C**, **D** Sigma-2/PGRMC1 agonists siramesine, WC-26 and SV-119 cause dose-dependent cell death in primary hippocampal/cortical cultures (**C**) and in SKOV-3 human ovarian cancer cells (**D**) but sigma-2/PGRMC1 antagonists RHM-1, CT0109 and CT0093 do not, except at very high concentrations (>100 µM). (**E**) Treatment of cultures of hippocampal and cortical cells with 20 to 80 µM SV-119 for 24 hours induced the activation of caspase 3/7 (*p<0.05 by 2-tailed Student's t-test compared to control). Co-treatment of cultures with 40 µM CT0109 or CT0093 did not increase caspase activity and blocked the activation by the agonist SV-119.

Little is known about the functional pharmacology of ligands for the sigma-2/PGRMC1 receptor. In tumor cells, agonists for sigma-2/PGRMC1 cause toxicity by triggering caspase 3 activation [46],[47] whereas sigma-2/PGRMC1 antagonists do not [47],[48]. We tested the behaviorally efficacious compounds CT0109 and CT0093 [30] to determine whether they behave as functional agonists or antagonists. In both primary hippocampal/cortical cultures and in the SKOV-3 tumor cell line, sigma-2/PGRMC1 agonist siramesine activated caspase-3/7 (**Fig. 8A, B**) while CT0109, CT0093 and the sigma-2 antagonist RHM-1 did not. Similarly, sigma-2/PGRMC1 agonists siramesine, WC-26 and SV-119 caused dose-dependent cell death in mature primary neuronal cultures (**Fig. 8C**) and in SKOV-3 human ovarian cancer cells (**Fig. 8D**), measured using fluorescent substrates for caspase-3/7 to detect enzymatic activity, but sigma-2/PGRMC1 antagonists RHM-1, CT0109 and CT0093 did not affect cell viability except at very high concentrations (>100 µM). These results indicate that the compounds CT0093 and CT0109 behave as antagonists in these functional assays. Finally, we treated hippocampal and cortical cultures with ascending concentrations of SV119 to induce caspase activation, in the presence of 40 µM of each of the sigma-2/PGRMC1 antagonists CT0109, CT0093 and RHM-1 (**Fig. 8E**). CT0109 and CT0093 significantly blocked the activation of caspase-3/7 by SV119, but the antagonist RHM-1 did not. This result suggests that there may be functionally distinct subtypes of sigma-2/PGRMC1 antagonist.

## Discussion

Synaptic dysfunction and loss caused by age-dependent accumulation of synaptotoxic Amyloid beta (Abeta) 1–42 oligomers has been proposed to underlie cognitive decline in Alzheimer's disease (AD) [3]–[8],[49]. We have discovered highly brain penetrant small molecule receptor antagonists that rescue oligomer-induced synapse loss and membrane trafficking deficits *in vitro*, and cognitive deficits in Alzheimer's mouse models [30]. Counterscreening against a panel of receptors and ion channels revealed that these small molecules bind selectively and with high affinity to sigma-2/PGRMC1 receptors. Sigma-2/PGRMC1 receptors are expressed in neurons and glia at low levels in primary 21DIV mixed hippocampal and cortical cultures, and oligomer binding to neuronal puncta is positively correlated with sigma-2/PGRMC1 receptor expression. Abeta oligomer exposure progressively upregulates receptor expression *in vitro*, and perhaps in Alzheimer's disease as well. Sigma-2/PGRMC1 receptors are also expressed in human brain at low levels, and are dysregulated in Alzheimer's disease. Knock-down of sigma-2/PGRMC1 protein expression reduces oligomer binding up to 90%. CogRx's behaviorally efficacious small molecules appear to act as functional antagonists at the sigma-2/PGRMC1 receptor. These small molecules, as well as antibodies specific for the C-terminal region of sigma-2/PGRMC1 can prevent and competitively displace Abeta oligomer binding to neurons *in vitro*, and to human Alzheimer's patient brain sections in a dose-dependent manner. Collectively, these data demonstrate that Abeta oligomers bind directly to sigma-2/PGRMC1 receptors, or a protein closely associated with it that itself mediates oligomer binding. Regardless of the precise mechanism, sigma-2/PGRMC1 selective small molecule antagonists have the potential to be disease-modifying therapeutics for Alzheimer's disease patients.

Sigma-2/PGRMC1 translocates between subcellular and plasma membrane locations [33],[34],[50], and our studies suggest that the C-terminus of at least part of the plasma membrane-localized sigma-2/PGRMC1 protein appears to be exposed to the extracellular environment where it interacts directly with Abeta oligomers or a protein that directly binds them. While it has been suggested that the C-terminus of sigma-2/PGRMC1 is on the cytoplasmic side of cell surface membranes [50], there is evidence that this domain of the protein can be extracellular [51].

*In vitro*, the more sigma-2/PGRMC1 is expressed in cells, the more Abeta is bound to cells and treatment with Abeta oligomers results in a progressive upregulation of the amount of sigma-2/PGRMC1 expressed in cells. In the mammalian brain, sigma-2/PGRMC1 is normally expressed at low levels. In severely demented Alzheimer's disease patients (CDR stage 3) expected to have a high degree of cell loss, the density of sigma-2 receptors remains unchanged compared to age-matched normal individuals, while the density of the unrelated sigma-1 receptor is decreased by 54%, as has been previously reported [43]. Collectively, these data suggests that oligomers may upregulate expression levels of the receptors that mediate their own binding, contributing to disease pathology. The consequences of any potential receptor upregulation for eventual drug dosing in human clinical trials are not expected to be significant, as the total amount of drug present is typically in vast excess to the number of receptors expressed on tissue. Studies in cell lines have shown that binding affinity of drugs to sigma-2/PGRMC1 receptor is constant despite a 10-fold difference in expression levels (Bmax) of the receptors [52]. To date, there are no reports of mutations in sigma-2/PGRMC1 that are associated with neurodegenerative diseases, human but mutations in this receptor are very rare, suggesting this gene is essential. Antibodies specific for the C-terminus of this receptor disrupt the normal process of membrane trafficking within neurons and glia *in vitro*, reinforcing the concept that this protein is essential for normal function. It is therefore possible that this protein is critically involved in AD, but understanding its role will require clinical examination in a large cohort of patients.

There are several reports in the literature of Abeta oligomer receptors, but this is the first report we are aware of in which greater than 90% of Abeta oligomer binding could be abolished by genetic knock-down of the putative receptor. This is also the first report we are aware of demonstrating that oligomers can be competitively displaced from binding sites in dissociated cell culture and in tissue by small molecule therapeutic agents and antibodies specific for the same receptor. Using these compounds to calculate off-rate of endogenous human oligomers, as well as to investigate the equilibrium between the various structural forms of Abeta protein found in human and transgenic animal model brain tissue is the subject of future studies. Much has been made recently of variability in oligomer preparations [53], however, the fact that therapeutic compounds and antibodies displace both synthetic and endogenous human oligomers validates an approach in which multiple preparations are used and compared in the course of drug discovery and elucidation of the fundamental underpinnings of oligomer biology.

The compounds we identified as being capable of displacing the oligomers appear to act as functional antagonists at the sigma-2/PGRMC1 receptor because they can block small molecule agonist-induced caspase activation. If oligomers acted as agonists in this functional context (i.e., if they induced caspase activation), the story would be a simple one; oligomers trigger caspase-mediated signaling through sigma-2/PGRMC1 and antagonists block it. However, despite several literature reports that oligomers induce caspase activation [46],[54],[55] we have been unable to detect oligomer-induced caspase activation in 21 DIV primary hippocampal and cortical neuronal cultures following treatment for 3–48 hours at Abeta oligomer concentrations up to 28 uM. Therefore, rigorous designation of compounds as functional “agonists” and “antagonists” in the context of oligomer biology will await identification of relevant signaling pathways downstream of oligomer interactions with sigma-2/PGRMC1. Candidates proteins include Insig/SCAP, P4, MAP1LC3 and UVRAG, as well as those mediating cholesterol metabolism [33],[34]. Understanding the interaction of sigma-2/PGRMC1 with proteins involved in intracellular trafficking [56] may further explain the mechanisms by which Abeta oligomers exert an effect on membrane trafficking, synaptic plasticity and cognitive loss. It is interesting to note that even at the level of the well-described caspase activation downstream of sigma-2 selective ligand binding, not all functional antagonists appear to be equally effective. Relevant drug binding sites on sigma-2/PGRMC1 are still coming into focus. In the meantime, it is clear that the compounds described in this manuscript are capable of blocking the synaptotoxic effects of Abeta oligomers that occur via interactions with sigma-2/PGRMC1, and that they represent potential disease-modifying therapeutics.

### Pharmacological model for compound-receptor interactions

The accompanying paper [30] presented evidence supporting the hypothesis that our compounds block the synaptotoxic effects of Abeta oligomers via allosteric antagonism rather than direct competitive antagonism. The present results provide additional evidence favoring the former scenario. Direct competitive antagonism would not be expected to result in an enhanced decrease in prebound oligomers from tissue under non-equilibrium binding conditions. Instead the observed dose-dependent displacement of endogenous oligomers from human Alzheimer's brain tissue (**Fig. 7**) make it more likely that our compound changes the affinity of the receptor for Abeta oligomers and increases the off-rate of the oligomers. This experimental result also makes it unlikely that our compounds reduce oligomer binding by inducing internalization of oligomer receptors, since this machinery is not likely to be functioning in frozen tissue sections.

### Pharmacological model for oligomer-receptor interactions

The data presented here indicate that sigma-2/PGRMC1 is critically required for binding of Abeta oligomers to neurons, either as 1) a direct binding site, or 2) by modulating conformation of a direct binding site. In the first model, small molecule ligands for sigma-2/PGRMC1 could block the binding of Abeta oligomers by allosterically modulating sigma-2/PGRMC1 itself, and reduction of sigma-2/PGRMC1 protein expression by siRNA profoundly reduces oligomer binding by directly eliminating the binding site for Abeta. In the second model, small molecule ligands for sigma-2/PGRMC1 block the binding of Abeta oligomers by allosterically modulating sigma-2/PGRMC1, which in turn alters the conformation of a tightly associated oligomer receptor protein. Reduction of sigma-2/PGRMC1 protein expression by siRNA reduces oligomer binding by eliminating the ability of sigma-2/PGRMC1 to stabilize the binding conformation of the receptor protein. The data obtained to date are consistent with either model. Our compounds' mechanism of action is also consistent with both possibilities.

The saturable binding of oligomers to a single site on neurons, together with the total loss of oligomer binding in the presence of compound or antibody specific for sigma-2/PGRMC1, would seem to argue for the first model, however oligomer binding to another protein that is very tightly linked to sigma-2/PGRMC1 would show the same pattern. It could be argued that support for the second model comes from the fact that sigma-2/PGRMC1 receptors are ubiquitously distributed both within and outside the nervous system, yet oligomers only bind to a subset of neurons. The subcellular localization of sigma-2/PGRMC1 in different tissues and cell types, and how this localization may change in response to signaling or damage, is not currently clear. Additional support for the second model comes from published reports that PGRMC1 stabilizes plasma membrane localization of EGFR and mPR*α* [57],[58].

Some support for a model with PGRMC1 modulating a binding site for Abeta oligomers could come from the lack of a 1∶1 stoichiometric relationship between loss of oligomer binding and loss of PGRMC1 protein expression as detected by immunohistochemistry (**Fig. 4**). Reduction of sigma-2/PGRMC1 expression levels of up to 30% in neurons leads to reduction of oligomer binding up to 91%. The two are highly correlated (r^2^ = 0.799 for cell bodies and r^2^ = 0.554 for synaptic puncta) with a slope of 2.5±0.5 for correlations with cell bodies and 2.6±0.5 for correlation with synaptic puncta, suggesting that for every one PGRMC1 receptor lost, 2–3 oligomers are not binding. This data suggests that the relationship between sigma-2/PGRMC1 expression and Abeta oligomer binding may not be strictly stoichiometric (one sigma-2/PGRMC1 molecule may bind to three oligomers, or one PGRMC1 may coordinately regulate expression and/or modulate three molecules which themselves bind Abeta oligomers directly). The details of this stoichiometry would need to be examined with quantitative labeling techniques for both sigma-2/PGRMC1 and Abeta oligomers, which is currently quite challenging to perform on oligomers without knowing whether such modifications alter secondary and tertiary structure. Whether this would be the case for human oligomers in tissue sections remains to be seen, and would require identification of equilibrium binding conditions and an understanding of the stoichiometry between detection antibodies and human oligomers. The lack of stoichiometry could also be due to a differential effect of PGRMC1 protein loss on Abeta oligomer binding to a subset of the neuronal population; the two possibilities are not mutually exclusive.

The second model is also supported by reports of oligomer receptor candidates that have appeared in the literature [9]–[11],[13],[27]–[29]. For several of these candidate receptors, oligomer binding to neurons (visualized via immunohistochemistry) is reduced by at most 50% when the receptor levels are eliminated by genetic knock-out [27],[29]. In contrast, siRNA-mediated reduction of sigma-2/PGRMC1 results in greater than 90% reduction of oligomer binding with a strong correlation between the amount of knock down of sigma-2/PGRMC1 protein and the amount of reduction of oligomer binding (r^2^ = 0.7994 for cell bodies and r^2^ = 0.554 for synaptic puncta). Small molecules selective for sigma-2/PGRMC1 completely eliminate detectable oligomer binding from neurons *in vitro* and dose-dependently displace oligomers from Alzheimer's patient brain tissue. If oligomers do bind directly to another protein, sigma-2/PGRMC1 likely plays a central role in its regulation. This is supported by PGRMC1's demonstrated role in stabilizing surface expression of transmembrane receptors [57],[58]. Additional studies will be required to test whether there is a direct interaction of sigma-2/PGRMC1 with candidate oligomer receptors. Given what is known about the mechanisms of synaptic plasticity, it is possible that the single, saturable oligomer binding site is in fact part of a multi-protein receptor complex that may include several of these candidate receptors. Such a multi-protein complex might change its constituent proteins depending on the electrical signaling pattern and history at a given synapse and thus exhibit a state-dependency. In contrast to other candidate oligomer receptors, small molecules with high affinity for sigma-2 receptors have reached Phase II clinical trials with no apparent mechanism of action-based toxicity reports. Decades of study have implicated sigma-2 receptors in several CNS disorders, but it has not been previously implicated in Alzheimer's disease or other neurodegenerative disorders. To date, no clinical trials with selective small molecules targeting this receptor for treatment of Alzheimer's disease have been conducted. The identification of the sigma-2 ligand binding activity as the protein PGRMC1 has opened up new avenues of research into CNS damage and dysregulation, and the mechanisms that mediate it [32].

Abeta oligomers are formed via self-association of monomeric protein that builds up with age, and are likely to be structurally polydisperse in the brain. Oligomer levels correlate with cognitive function in Alzheimer's disease [6],[59],[60]. Our evidence indicates that oligomers bind saturably to a single binding site on neurons and can be displaced by small molecule allosteric antagonists. We have also shown that oligomers bind directly to sigma-2/PGRMC1 or a protein whose binding site for oligomers can be modulated by sigma-2/PGRMC1. The high affinity, selective sigma-2/PGRMC1 compounds that we have discovered are capable of preventing binding of oligomers and of displacing bound oligomers, thereby preventing and treating oligomer-induced trafficking deficits and synapse regression. These molecules rapidly prevent and treat cognitive deficits in wild-type and aged transgenic Alzheimer's mouse models and sustain this cognitive improvement long term [30].

Oligomers thus represent pathological ligands that behave according to the principles of mass action. Brain-penetrant antagonists to sigma-2/PGRMC1 receptors represent a novel approach to blocking Abeta oligomer binding and downstream signaling and are potentially capable of halting disease progression. This is good news for Alzheimer's patients, for whom no disease-modifying therapies currently exist.

## Supporting Information

Figure S1 **PGRMC1's C-terminal amino acids 185–195 are exposed at the extracellular surface of the plasma membrane.** Untreated cultures were formaldehyde-fixed then immunolabeled with anti-synaptophysin antibody. This antibody can only detect the synaptophysin protein following detergent permeabilization of the plasma membrane (**A**), which allows the large IgG molecule physical access to the intracellularly located synaptophysin protein. In the absence of detergent, punctate synaptophysin immunolabeling is not visible (**B**). In contrast, cultures immunolabeled with anti-PGRMC1 antibody directed against the protein's C-terminal amino acids 185–195 can detect PGRMC1 in the absence of detergent (**D**), although it is 55%±7 (S.D.) less intense than that which is detected following permeabilization with detergent (**C**), indicating that this region of the protein is not located exclusively intracellularly. This suggests that substantial amounts of the C-terminus are likely exposed on the plasma membrane extracellular surface. Scale bar  = 20 microns.
(TIF)

Figure S2 **siRNA-mediated reduction of PGRMC1 expression reduces the number of neurons that exhibit the most intense binding of Abeta oligomers.** **(Note different y-axis scales) A–D** siRNA-treated cultures (black bars) exhibit fewer cells labeled most brightly with Abeta oligomers compared to untreated cultures; these neurons also have the highest sigma-2/PGRMC1 expression. Dividing the cell population into Abeta oligomer binding intensity bins allows this absence to be seen quantitatively. siRNA-treated neurons (filled bars) have similar numbers of neurons as untreated cultures (open bars, **A**), and similar numbers of neurons with little (**B**) or moderate (**C**) detectable Abeta oligomer binding to neuritic puncta, but exhibit a dramatic absence of the most brightly oligomer-labeled neurons expressing the highest levels of sigma-2/PGRMC1 protein (**D**) compared to untreated cultures. In untreated cultures (open bars), neurons with Abeta oligomer punctate labeling of >300 average intensity (**D**) represent 27% of the total neuronal population (**A**). Following siRNA treatment (black bars), this neuronal population decreases to 3% of total. Thus the impact on this bright binding population may have a disproportionate effect on the population total binding average. This is one possible reason why siRNA-mediated reduction of PGRMC1 protein expression by 30% but reduces Abeta oligomer binding by 90%.
(TIF)

Figure S3 **MAPR family sequence conservation across species.**
(DOCX)

Figure S4 **Method of analyzing endogenous Abeta oligomer binding displacement from fresh frozen post-mortem neocortical Alzheimer's patient brain sections.** **A**. Brain tissue section showing ThioS labeling of dense core plaques and (**B**) same section immunolabeled for Abeta 1–42. **C**, **D** Enlargement of yellow boxes in **A** and **B** showing individual plaques (**C**) and corresponding Abeta labeling (**D**). **E**, **F** Enlargement showing single plaques and Abeta label. **G**. Outline of mask drawn around one plaque and 2 µm plaque halo around edge of plaque by analysis macro. **H** Mask is transferred to Abeta immuno-fluorescent channel and intensity in the plaque halos are measured. **I**. Table shows characteristics of patients with a diagnosis of AD (CERAD score “definite” by postmortem neuropathological exam) used in this study and number of plaques analyzed in each treatment group from each case. Statistical analysis of data from this experiment is described in Methods.
(TIF)

Table S1 **Activity of CT0109 in target screening panel.**
(DOCX)

Table S2 **Genetic analysis of MAPR family members PGRMC1, PGRMC2, neudesin (NENF) and neuferricin.**
(DOCX)
