# Supplementary figures and images for: Alzheimer's Therapeutics Targeting Amyloid Beta 1–42 Oligomers II: Sigma-2/PGRMC1 Receptors Mediate Abeta 42 Oligomer Binding and Synaptotoxicity
Source: PLoS One. 2014 Nov 12;9(11):e111899. doi: 10.1371/journal.pone.0111899 (PMC4229119; doi:10.1371/journal.pone.0111899)

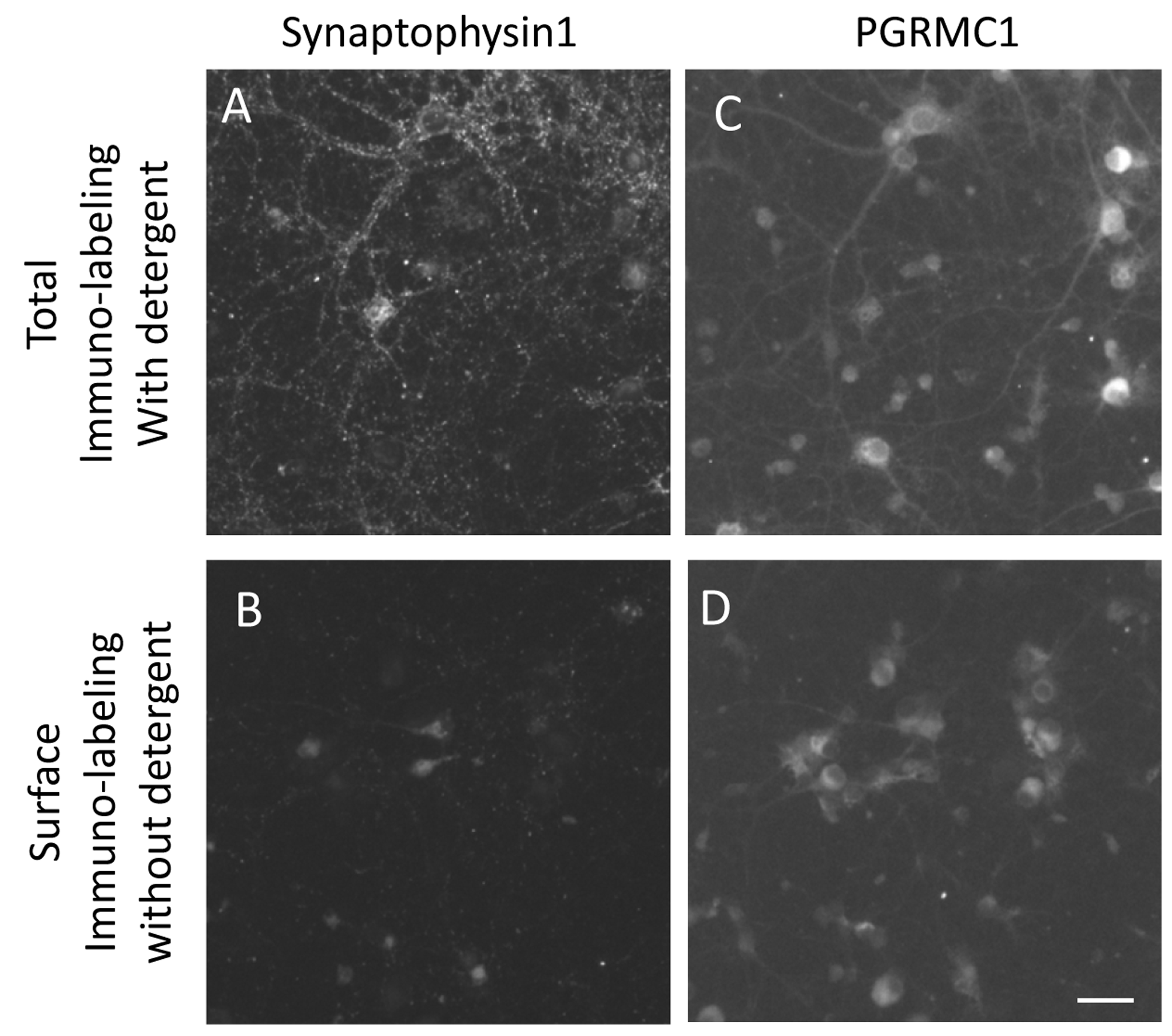

Supplement: Figure S1 — PGRMC1's C-terminal amino acids 185–195 are exposed at the extracellular surface of the plasma membrane. Untreated cultures were formaldehyde-fixed then immunolabeled with anti-synaptophysin antibody. This antibody can only detect the synaptophysin protein following detergent permeabilization of the plasma membrane (A), which allows the large IgG molecule physical access to the intracellularly located synaptophysin protein. In the absence of detergent, punctate synaptophysin immunolabeling is not visible (B). In contrast, cultures immunolabeled with anti-PGRMC1 antibody directed against the protein's C-terminal amino acids 185–195 can detect PGRMC1 in the absence of detergent (D), although it is 55%±7 (S.D.) less intense than that which is detected following permeabilization with detergent (C), indicating that this region of the protein is not located exclusively intracellularly. This suggests that substantial amounts of the C-terminus are likely exposed on the plasma membrane extracellular surface. Scale bar = 20 microns. (TIF) [file pone.0111899.s001.tif]

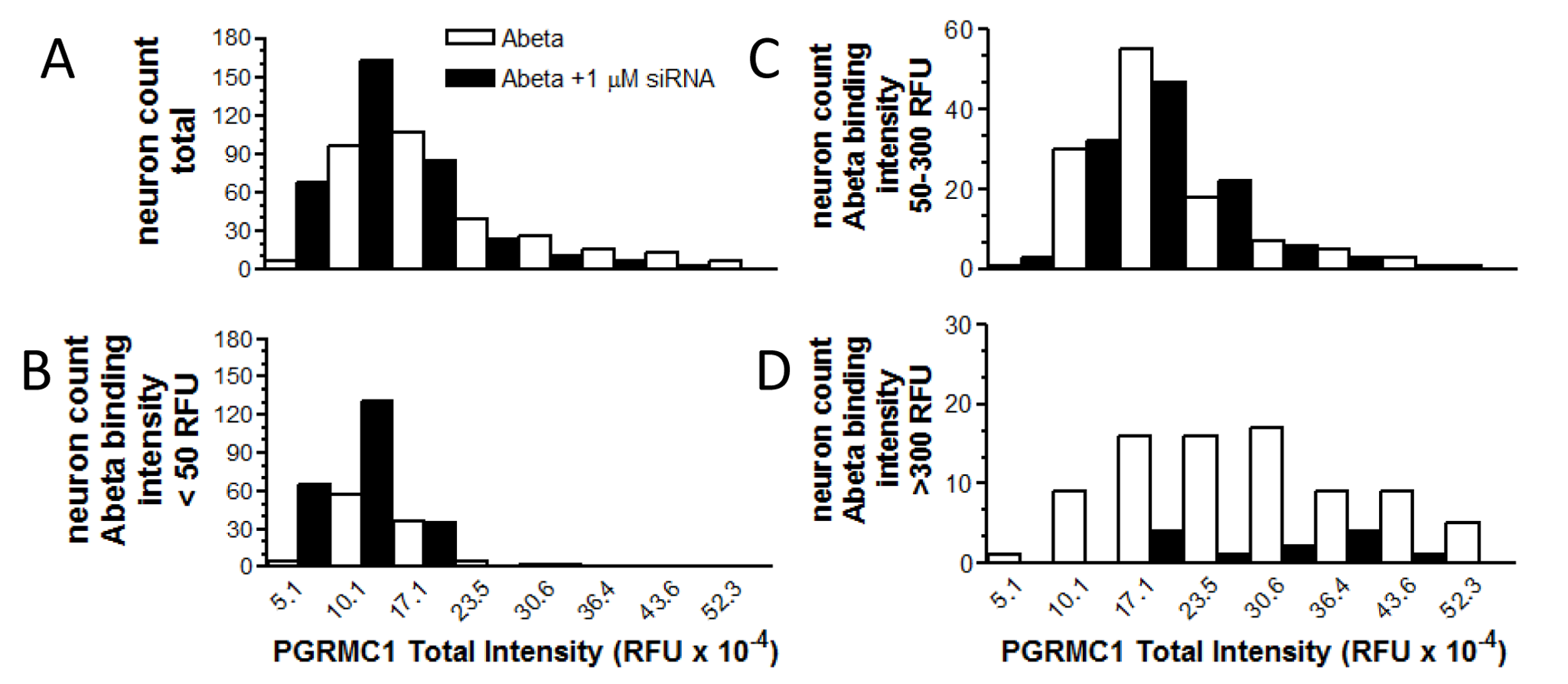

Supplement: Figure S2 — siRNA-mediated reduction of PGRMC1 expression reduces the number of neurons that exhibit the most intense binding of Abeta oligomers. (Note different y-axis scales) A–D siRNA-treated cultures (black bars) exhibit fewer cells labeled most brightly with Abeta oligomers compared to untreated cultures; these neurons also have the highest sigma-2/PGRMC1 expression. Dividing the cell population into Abeta oligomer binding intensity bins allows this absence to be seen quantitatively. siRNA-treated neurons (filled bars) have similar numbers of neurons as untreated cultures (open bars, A), and similar numbers of neurons with little (B) or moderate (C) detectable Abeta oligomer binding to neuritic puncta, but exhibit a dramatic absence of the most brightly oligomer-labeled neurons expressing the highest levels of sigma-2/PGRMC1 protein (D) compared to untreated cultures. In untreated cultures (open bars), neurons with Abeta oligomer punctate labeling of >300 average intensity (D) represent 27% of the total neuronal population (A). Following siRNA treatment (black bars), this neuronal population decreases to 3% of total. Thus the impact on this bright binding population may have a disproportionate effect on the population total binding average. This is one possible reason why siRNA-mediated reduction of PGRMC1 protein expression by 30% but reduces Abeta oligomer binding by 90%. (TIF) [file pone.0111899.s002.tif]

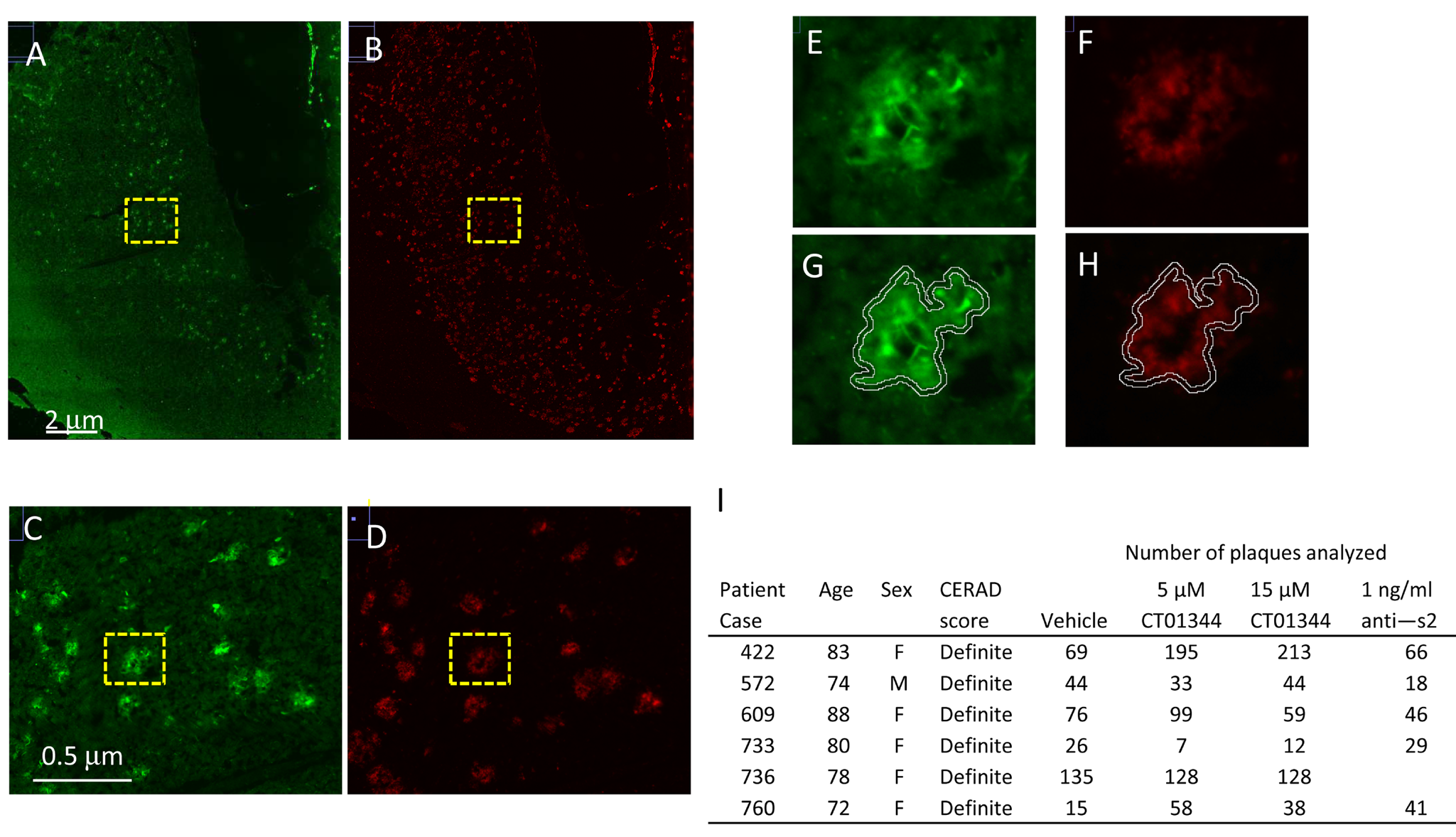

Supplement: Figure S4 — Method of analyzing endogenous Abeta oligomer binding displacement from fresh frozen post-mortem neocortical Alzheimer's patient brain sections. A. Brain tissue section showing ThioS labeling of dense core plaques and (B) same section immunolabeled for Abeta 1–42. C, D Enlargement of yellow boxes in A and B showing individual plaques (C) and corresponding Abeta labeling (D). E, F Enlargement showing single plaques and Abeta label. G. Outline of mask drawn around one plaque and 2 µm plaque halo around edge of plaque by analysis macro. H Mask is transferred to Abeta immuno-fluorescent channel and intensity in the plaque halos are measured. I. Table shows characteristics of patients with a diagnosis of AD (CERAD score “definite” by postmortem neuropathological exam) used in this study and number of plaques analyzed in each treatment group from each case. Statistical analysis of data from this experiment is described in Methods. (TIF) [file pone.0111899.s004.tif]
