# Supplementary material for: Alzheimer's Therapeutics Targeting Amyloid Beta 1–42 Oligomers II: Sigma-2/PGRMC1 Receptors Mediate Abeta 42 Oligomer Binding and Synaptotoxicity
Source: PLoS One. 2014 Nov 12;9(11):e111899. doi: 10.1371/journal.pone.0111899 (PMC4229119; doi:10.1371/journal.pone.0111899)
Supplement: Figure S3 — MAPR family sequence conservation across species. (DOCX) [file pone.0111899.s003.docx]

**A) PGRMC1 alignment with human MAPR proteins**


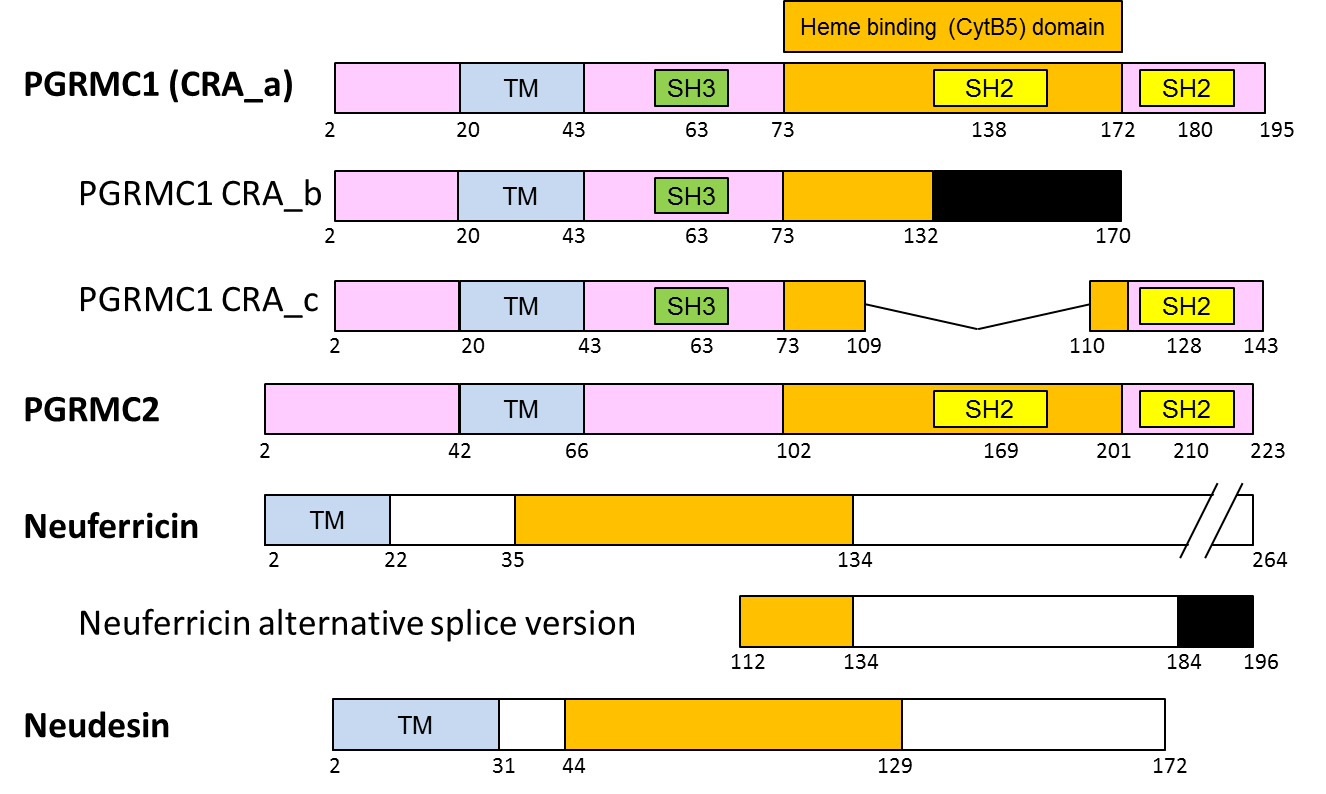


**B) PGRMC1 vertebrate species alignment**

O00264 PGRC1_HUMAN 1 MAAEDVVATGADPSDLESGGLLHEIFTSPLNLLLLGLCIFLLYKIVRGDQPAASGDSDDD 60

K6ZK25 K6ZK25_PANTR 1 MAAEDVVATGADPSDLESGGLLHEIFTSPLNLLLLGLCIFLLYKIVRGDQPAASGDSDDD 60

F7BA07 F7BA07_MACMU 1 MAAEDAVATGADPSELESGGLLHEIFTSPLNLLLLGLCIFLLYKIVRGDQPAASGDSDDD 60

O55022 PGRC1_MOUSE 1 MAAEDVVATGADPSELEGGGLLHEIFTSPLNLLLLGLCIFLLYKIVRGDQPGASGDNDDD 60

P70580 PGRC1_RAT 1 MAAEDVVATGADPSELEGGGLLQEIFTSPLNLLLLGLCIFLLYKIVRGDQPGASGDNDDD 60

Q17QC0 PGRC1_BOVIN 1 MAAEDVAATGADTSELESGGLLQEIFTSPLNLLLLGLCIFLLYKIVRGDQPAAS-DSDDD 59

E2RJW8 E2RJW8_CANFA 1 MAAEDVAATGADPSELEGGGLLHEIFTSPLNLLLLGLCVFLLYKIVRGDQPAAGGDSDDD 60

Q5ZKN2 PGRC1_CHICK 1 MAAEEPAMAGEEAVATEGGGLLLEIVGSPLNLSLLGLCLFLLYQILRGERPAAQ--PGEA 58

Q6GQL5 Q6GQL5_XENLA 1 MA-EE--------------GILQEIFTSPLNICLLCLCLYLLYKILRGDKPQSNENT--- 42

Q5U3G0 Q5U3G0_DANRE 1 MA-EEA-------VE-QTSGILQEIFTSPLNISLLCLCLFLLYKIIRGDKPADYGPV--- 48

** *: *:* **. ****: ** **::***:*:**::*

O00264 PGRC1_HUMAN 61 EPPPLPRLKRRDFTPAELRRFDGVQDPRILMAINGKVFDVTKGRKFYGPEGPYGVFAGRD 120

K6ZK25 K6ZK25_PANTR 61 EPPPLPRLKRRDFTPAELRRFDGVQDPRILMAINGKVFDVTKGRKFYGPEGPYGVFAGRD 120

F7BA07 F7BA07_MACMU 61 EPPPLPRLKRRDFTPAELRRFDGVQDPRILMAINGKVFDVTKGRKFYGPEGPYGVFAGRD 120

O55022 PGRC1_MOUSE 61 EPPPLPRLKRRDFTPAELRRFDGVQDPRILMAINGKVFDVTKGRKFYGPEGPYGVFAGRD 120

P70580 PGRC1_RAT 61 EPPPLPRLKPRDFTPAELRRYDGVQDPRILMAINGKVFDVTKGRKFYGPEGPYGVFAGRD 120

Q17QC0 PGRC1_BOVIN 60 EPPPLPRLKRRDFTPAELRRFDGVQDPRILMAINGKVFDVTKGRKFYGPEGPYGVFAGRD 119

E2RJW8 E2RJW8_CANFA 61 EPPPLPRLKRRDFTPAELRRFDGVQDPRILMAINGKVFDVTKGRKFYGPEGPYGVFAGRD 120

Q5ZKN2 PGRC1_CHICK 59 GPPPLPKMKRRDFTLEQLRPYDGVRDPRILMAVNGKVFDVTRASKFYGPDGPYGIFAGRD 118

Q6GQL5 Q6GQL5_XENLA 43 -EEQLPKMKKRDFTRAELQEYDGVQNPRILMAISNKVFDVTRGKKFYGPDGPYGIFAGRD 101

Q5U3G0 Q5U3G0_DANRE 49 -EEPLPKLKKRDFTLADLQEYDGLKNPRILMAVNGKVFDVTRGKKFYGPEGPYGVFAGKD 107

**::* **** :*: :**:::******:. ******:. *****:****:***:*

O00264 PGRC1_HUMAN 121 ASRGLATFCLDKEALKDEYDDLSDLTAAQQETLSDWESQFTFKYHHVGKLLKEGEEPTVY 180

K6ZK25 K6ZK25_PANTR 121 ASRGLATFCLDKEALKDEYDDLSDLTAAQQETLSDWESQFTFKYHHVGKLLKEGEEPTVY 180

F7BA07 F7BA07_MACMU 121 ASRGLATFCLDKEALKDEYDDLSDLTAAQQETLSDWESQFTFKYHHVGKLLKEGEEPTVY 180

O55022 PGRC1_MOUSE 121 ASRGLATFCLDKEALKDEYDDLSDLTPAQQETLSDWDSQFTFKYHHVGKLLKEGEEPTVY 180

P70580 PGRC1_RAT 121 ASRGLATFCLDKEALKDEYDDLSDLTPAQQETLNDWDSQFTFKYHHVGKLLKEGEEPTVY 180

Q17QC0 PGRC1_BOVIN 120 ASRGLATFCLDKEALKDEYDDLSDLTPAQQETLSDWDSQFTFKYHHVGKLLKDGEEPTVY 179

E2RJW8 E2RJW8_CANFA 121 ASRGLATFCLDKEALKEEYDDLSDLTPAQQETLSDWDSQFTFKYHHVGKLLKEGEEPTVY 180

Q5ZKN2 PGRC1_CHICK 119 ASRGLATFCLDKEALRDDYDDLSDLNATQQETLRDWESQFTFKYHHVGKLLKDGEEPTVY 178

Q6GQL5 Q6GQL5_XENLA 102 ASRGLATFCLDKDALKDTDDDLSDLTATQKETLNDWEEQFTFKYHHVGKLLKAGEEPTEY 161

Q5U3G0 Q5U3G0_DANRE 108 ASRGLATFCLEKEALKDTHDDLSDLNAMQQESLSEWETQFTQKYDYIGKLLKPGEEPTEY 167

**********:*:**:: ******. *:*:* :*: *** **.::***** ***** *

O00264 PGRC1_HUMAN 181 SDEEEPKDESARKND- 195

K6ZK25 K6ZK25_PANTR 181 SDEEEPKDESARKND- 195

F7BA07 F7BA07_MACMU 181 SDEEEPKDESARKND- 195

O55022 PGRC1_MOUSE 181 SDDEEPKDETARKNE- 195

P70580 PGRC1_RAT 181 SDDEEPKDEAARKSD- 195

Q17QC0 PGRC1_BOVIN 180 SDKEEPKDESTRKND- 194

E2RJW8 E2RJW8_CANFA 181 SDEEEAKDENARKND- 195

Q5ZKN2 PGRC1_CHICK 179 SDEEEKDAQDAKKE-- 192

Q6GQL5 Q6GQL5_XENLA 162 TDDEDAKDSSDSKKKN 177

Q5U3G0 Q5U3G0_DANRE 168 TDDEEVKDKKKD---- 179

:*.*: . .

**C) PGRMC2 vertebrate species alignment**

O15173 PGRC2_HUMAN 1 ------------------------MAAGDGDVKLGTLGSGSESSNDGGSESPGDAGAAAE 36

H2QQ58 H2QQ58_PANTR 1 MGGAARGVGEGRGRGGGGRRWRAVMAAGDGDVKLGTLGSGSESSNDGGSESPGGAGAAAE 60

F7GRJ8 F7GRJ8_MACMU 1 ------------------------MAAGDGDVKLGTLGSGSESSSDGGSESPGGAGAAAE 36

Q80UU9 PGRC2_MOUSE 1 ------------------------MAAGDGDVKLSTLGSGGESGGD---GSPGGAGATAA 33

Q5XIU9 PGRC2_RAT 1 ------------------------MAAGDGDVKLSTLGSGGERGGD---GSPGGAGATAA 33

A5PJQ6 A5PJQ6_BOVIN 1 ------------------------MAAGDGDVNLGTLGSGSESSSDGSSESPGGAGAAAE 36

F1PN69 F1PN69_CANFA 1 ------------------------MAAGDGDVKLGTLGSGSESSSDGSSESPSGVGAAAE 36

Q5ZLX0 Q5ZLX0_CHICK 1 -----------------------MADGGDG--RLRT----AESGGDGGAGE--------- 22

Q66IX1 Q66IX1_XENLA 1 ----------------MSESWE--------------------VEEQLAGTSPPGREGEPS 24

Q7SZC7 Q7SZC7_DANRE 1 -----------------------MADDGDGRSAVA------DTSGDQGTTEEL------- 24

: .

O15173 PGRC2_HUMAN 37 GGGWAAAALALLTGGGEMLLNVALVALVLLGAYRLWVRWGRRGLGAGAGAGEESPATSLP 96

H2QQ58 H2QQ58_PANTR 61 GGGWAAAALALLTGGGEMLLNVALVALMLLGAYRLWVRWGRRGLGAGAGAGEESPAASLP 120

F7GRJ8 F7GRJ8_MACMU 37 GGGWAAAALALLTGGGEMLLNVALVALVLLGAYRLWVRWGRRGLGTGAGAGEESPAASLP 96

Q80UU9 PGRC2_MOUSE 34 RSSWVAA---LLATGGEMLLNVALVALVLLGAYRLWVRWGRRGLCSGPGAGEESPAATLP 90

Q5XIU9 PGRC2_RAT 34 RSSWVAA---LLATGGEMLLNVALVALVLLGAYRLWVRWGRRGLCSGPGAGEESPAATLP 90

A5PJQ6 A5PJQ6_BOVIN 37 GGSCLATALALLTGGGEMLLNVALVALVLLGAYRLWVRWGRRGLGAGAGAGEESPAASLP 96

F1PN69 F1PN69_CANFA 37 GGGWAAAALALLTGGGEMLLNVALVALVLLGAYRLWVRWGRRGLGTGAGAGEESPAASLP 96

Q5ZLX0 Q5ZLX0_CHICK 23 ------------PAGGGMLLHVGLLALVLLAAYRLYLRWRKRSALG--GAAQQSQAALLP 68

Q66IX1 Q66IX1_XENLA 25 GLGW--------LFVGELLLNAALVLVLIYGAFRIYQRWKGSGSG---------GAASLP 67

Q7SZC7 Q7SZC7_DANRE 25 -------DPGAGLGLGGMLLNLSVLVLVLAACYVLYARWWRRA---GADLGRGSEASPLP 74

* :**. .:: ::: ..: :: ** . *: **

O15173 PGRC2_HUMAN 97 RMKKRDFSLEQLRQYDGSRNPRILLAVNGKVFDVTKGSKFYGPAGPYGIFAGRDASRGLA 156

H2QQ58 H2QQ58_PANTR 121 RMKKRDFSLEQLRQYDGSRNPRILLAVNGKVFDVTKGSKFYGPAGPYGIFAGRDASRGLA 180

F7GRJ8 F7GRJ8_MACMU 97 RMKKRDFSLEQLRQYDGSRNPRILLAVNGKVFDVTKGSKFYGPAGPYGIFAGRDASRGLA 156

Q80UU9 PGRC2_MOUSE 91 RMKKRDFSLEQLRQYDGARTPRILLAVNGKVFDVTKGSKFYGPAGPYGIFAGRDASRGLA 150

Q5XIU9 PGRC2_RAT 91 RMKKRDFSLEQLRQYDGARTPRILLAVNGKVFDVTKGSKFYGPAGPYGIFAGRDASRGLA 150

A5PJQ6 A5PJQ6_BOVIN 97 RMKKRDFSLEQLRQYDGSRNPRILLAVNGKVFDVTKGSKFYGPAGPYGIFAGRDASRGLA 156

F1PN69 F1PN69_CANFA 97 RMKKRDFSLEQLRQYDGSRTPRILLAVNGKVFDVTKGSKFYGPAGPYGIFAGRDASRGLA 156

Q5ZLX0 Q5ZLX0_CHICK 69 RMKRRDFSLEQLREFDGTRNPRILLAVNGKVFDVTKGSKFYGPEGPYGIFAGRDASRGLA 128

Q66IX1 Q66IX1_XENLA 68 RMKRRDFTLQQLREYDGTHKPRILLAVNGKVFDVTQGSKSYGPDGPYGLFAGRDASRGLA 127

Q7SZC7 Q7SZC7_DANRE 75 KMRRRDFTLQQLRDYDGVQNPRILMAVNTKVFDVTSGKKFYGREGPYGIFAGRDASRGLA 134

:*::***:*:***::** :.****:*** ******.*.* ** ****:***********

O15173 PGRC2_HUMAN 157 TFCLDKDALRDEYDDLSDLNAVQMESVREWEMQFKEKYDYVGRLLKPGEEPSEYTDEEDT 216

H2QQ58 H2QQ58_PANTR 181 TFCLDKDALRDEYDDLSDLNAVQMESVREWEMQFKEKYDYVGRLLKPGEEPSEYTDEEDT 240

F7GRJ8 F7GRJ8_MACMU 157 TFCLDKDALRDEYDDLSDLNAVQMESVREWEMQFKEKYDYVGRLLKPGEEPSEYTDEEDT 216

Q80UU9 PGRC2_MOUSE 151 TFCLDKDALRDEYDDLSDLNAVQMESVREWEMQFKEKYDYVGRLLKPGEEPSEYTDEEDT 210

Q5XIU9 PGRC2_RAT 151 TFCLDKDALRDEYDDLSDLNAVQMESVREWEMQFKEKYDYVGRLLKPGEEPSEYTDEEDT 210

A5PJQ6 A5PJQ6_BOVIN 157 TFCLDKDALKDEYDDLSDLNAVQMESVREWEMQFKEKYDYVGRLLKPGEEPSEYTDEEDT 216

F1PN69 F1PN69_CANFA 157 TFCLDKDALKDEYDDLSDLNAVQMESVREWEMQFKEKYDYVGRLLKPGEEPSEYTDEEDT 216

Q5ZLX0 Q5ZLX0_CHICK 129 TFCLDKDALRDEYDDLSDLNAVQMESVREWEMQFKEKYDYVGRLLKPGEEPSEYTDEEDT 188

Q66IX1 Q66IX1_XENLA 128 TFCLDKEALRDEYDDLSDLNAVQMESVREWEMQFKDKYEYVGRLLKPGEEPSEYTDEEDV 187

Q7SZC7 Q7SZC7_DANRE 135 TFCLEKDALRDEYDDLSDLNAVQMESVREWEMQFMEKYDYVGRLLKPGDEPSEYTDEEDM 194

****:*:**:************************ :**:*********:**********

O15173 PGRC2_HUMAN 217 KDHNKQD 223

H2QQ58 H2QQ58_PANTR 241 KDHNKQD 247

F7GRJ8 F7GRJ8_MACMU 217 KDHNKQD 223

Q80UU9 PGRC2_MOUSE 211 KDHSKQD 217

Q5XIU9 PGRC2_RAT 211 KDHSKQD 217

A5PJQ6 A5PJQ6_BOVIN 217 KDHNKQD 223

F1PN69 F1PN69_CANFA 217 KDHNKQD 223

Q5ZLX0 Q5ZLX0_CHICK 189 KDHTKQE 195

Q66IX1 Q66IX1_XENLA 188 RDHTKQD 194

Q7SZC7 Q7SZC7_DANRE 195 KDHQKHE 201

:** *::

**D) PGRMC1 v. PGRMC2 human alignment**

O00264 PGRC1_HUMAN 1 MAAED-------------------VVATGADPSDLESGGLLHEI---F----TSPLNLLL 34 O15173 PGRC2_HUMAN 1 MAAGDGDVKLGTLGSGSESSNDGGSESPGDAGAAAEGGGWAAAALALLTGGGEMLLNVAL 60

*** * . : * : *.** : **: *

O00264 PGRC1_HUMAN 35 LGLCIFLLYK----IVRGDQPAASGDSDDDEPPPLPRLKRRDFTPAELRRFDGVQDPRIL 90 O15173 PGRC2_HUMAN 61 VALVLLGAYRLWVRWGRRGLGAGAGAGEESPATSLPRMKKRDFSLEQLRQYDGSRNPRIL 120

:.* :: *: * *.:* .::. ***:*:***: :**::** ::****

O00264 PGRC1_HUMAN 91 MAINGKVFDVTKGRKFYGPEGPYGVFAGRDASRGLATFCLDKEALKDEYDDLSDLTAAQQ 150 O15173 PGRC2_HUMAN 121 LAVNGKVFDVTKGSKFYGPAGPYGIFAGRDASRGLATFCLDKDALRDEYDDLSDLNAVQM 180

:*:********** ***** ****:*****************:**:*********.*.*

O00264 PGRC1_HUMAN 151 ETLSDWESQFTFKYHHVGKLLKEGEEPTVYSDEEEPKDESARKND 195

O15173 PGRC2_HUMAN 181 ESVREWEMQFKEKYDYVGRLLKPGEEPSEYTDEEDTKDHN--KQD 223

*:: :** **. **.:**:*** ****: *:***: **.. *:*

**Figure S3.** Primary structure of PGRMC1-related proteins, and sequence conservation of vertebrate PGRMC1. **A.** The schematic primary isoform of human PGRMC1 (CRA_a) is aligned with predicted PGRMC1 isoforms CRA_b and CRA_C, as well as the other Membrane Associated Progesterone Receptor (MAPR) family members including PGRMC2, two predicted splice variants of Neuferricin, and Neudesin. PGRMC1 CRA_b and CRA_c protein sequences are conceptual translations from nucleotide sequences. There is currently no report of their presence as actual proteins. Pink shading of PGRMC1 and PGRMC2 indicates their closely shared protein structure not shared with other MAPR proteins. Black boxes for PGRMC1 CRA_b and Neuferricin alternative splice version represent predicted amino acids that are not contained in an alternative isoform of the same protein. The conserved heme binding domain (related to cytochrome b5) common to all MAPR family members^2^ is boxed orange. Blue boxes correspond to the predicted transmembrane helix.

Other abbreviations are based on consensus predicted sequences only and have not been experimentally verified: “SH3” (green boxes) and “SH2” (yellow boxes) denote target sequences for binding of SH3 and SH2 domain proteins to PGRMC1^1^ and corresponding conserved sequences in PGRMC2. Numbers below the proteins depict the amino acid numbers from the following protein sequences: PGRMC1 CRA_a (UniProt O00264, Genbank NP_006658, CCDS ID 14576.1); PGRMC1 CRA_b (Genbank EAW89880.1); PGRMC1 CRA_c (Genbank EAW89881); PGRMC2 (UniProt O15173); Neuferricin and Neuferricin alternative splice version (UniProt Q8WUJ1); Neudesin (UniProt Q9UMX5). The amino acid number associated with predicted SH2 target sequences for PGRMC1 and PGRMC2 denote the tyrosine residue that would have to be phosphorylated for interaction with SH2 domain proteins. Similarly, proline 63 is at the center of the predicted SH3 target sequence in PGRMC1^1^ that is not present in PGRMC2. A short amino acid loop inserted between helices 3 and 4 of the heme binding domain defines the MAPR family within the larger MAPR family.^1,2^ In PGRMC1^1^ and PGRMC2 this loop contains a tyrosine as the predicted target for SH2 proteins, absent in Neudesin and Neuferricin.

**B.** Protein sequence alignment of PGRMC1 from (*species;* with UniProt IDs) Human (*Homo sapiens;* O00264), Chimpanzee (*Pan Troglodytes;* K6ZK25), Rhesus Macaque (*Macaca mulatta;* F7BA07), Mouse (*Mus musculus;* O55022), Rat (*Rattus norvegicus;* P70580), Cow, (*Bos Taurus;* Q17QC0), Dog (*Canis familiaris;* E2RJW8), Chicken (*Gallus gallus;* Q5ZKN2), Frog (*Xenopus laevis;* Q6GQL5Z), and Zebrafish (*Danio rerio;* Q5U3G0). PGRMC1 amino acid sequences across species were aligned with the Clustal Omega^3^ multiple alignment tool on UniProt (http://www.uniprot.org/align/). The N-terminal alignments produced by Clustal Omega for Frog and Zebrafish were corrected manually to align the MA-EE motifs with the N-terminus. Amino acid numbers are given to the right of each line. **Alignment** **Key:** “–”amino acid not present in that species. “*” identical amino acid across all species. “: ” chemically similar amino acids across species. “.” conserved in all species except one.
**C.** Alignment of PGRMC2 protein sequences from the same species as **B**, using the same methods with the indicated Protein IDs from UniProt. Based upon the conserved PGRMC1 N-terminus in all other mammals, M25 is the most likely true initiator methionine of the Chimpanzee sequence. **D.** Alignment of human PGRMC1 and PGRMC2 sequences, as above. The Clustal Omega alignment was manually corrected to align D5 across both species.

**References**

1 Cahill, M. A. Progesterone receptor membrane component 1: an integrative review. *J Steroid Biochem Mol Biol* **105**, 16-36, (2007).

2 Mifsud, W. & Bateman, A. Membrane-bound progesterone receptors contain a cytochrome b5-like ligand-binding domain. *Genome Biol* **3**, RESEARCH0068, (2002).

3 Sievers, F. *et al.* Fast, scalable generation of high-quality protein multiple sequence alignments using Clustal Omega. *Molecular systems biology* **7**, 539, (2011).
