# Supplementary material for: Alzheimer's Therapeutics Targeting Amyloid Beta 1–42 Oligomers II: Sigma-2/PGRMC1 Receptors Mediate Abeta 42 Oligomer Binding and Synaptotoxicity
Source: PLoS One. 2014 Nov 12;9(11):e111899. doi: 10.1371/journal.pone.0111899 (PMC4229119; doi:10.1371/journal.pone.0111899)
Supplement: Table S1 — Activity of CT0109 in target screening panel. (DOCX) [file pone.0111899.s005.docx]

**Supplemental Table S1. Activity of CT0109 in target screening panel**

| **Target** | | **Cerep Catalog number** | | **Assay type** | | | **Binding Ki (nM)** | | | **EC50 (nM)** | | |
| --- | --- | --- | --- | --- | --- | --- | --- | --- | --- | --- | --- | --- |
| Sigma-2 | | 0148 | | Radioligand binding | | | 9 | | |  | | |
| Sigma-1 | | 0889 | | Radioligand binding | | | 500 | | |  | | |
| mu opioid | | 0118 | | Radioligand binding | | | 1700 | | |  | | |
| Na^+^ Channel (site2) | | 0169 | | Radioligand binding | | | 2000 | | |  | | |
| Dopamine 3 | | 0683 | | Agonist activity | | |  | | | 4100 | | |
| **Binding Activity > 10,000 nM** | | | | | | | | | | | | |
| **Target** | **Catalog No.** | | **Target** | | **Catalog No.** | | | **Target** | | | **Catalog No.** | |
| M1 | 091 | | NK3 | | 0104 | | | CAMK4 | | | 1582 | |
| M2 | 093 | | Y1 | | 0106 | | | CDK/p35 | | | 2877 | |
| M3 | 095 | | Y2 | | 0107 | | | EGFR kinase | | | 2865 | |
| Ca^+2^ Channel | 0163 | | NTS1 | | 0109 | | | EphA4 kinase | | | 1702 | |
| NE Transporter | 0355 | | Kapa opioid | | 1971 | | | EphB2 kinase | | | 3054 | |
| DA transporter | 0052 | | Delta 2 opioid | | 0114 | | | EphB4 kinase | | | 3059 | |
| 5-HT transporter | 0439 | | N Opioid | | 0358 | | | FAK | | | 3065 | |
| Adenosine Transporter | 0007 | | Sst | | 0149 | | | Choline Transporter | | | 1552 | |
| Adenosine 1 | 0002 | | VPAC1 | | 0157 | | | FGFR1 kinase | | | 2868 | |
| Adenosine A2A | 0004 | | V1a | | 0159 | | | FGFR3 kinase | | | 2894 | |
| AdenosineA3 | 0006 | | Kv channel | | 0166 | | | FGFR4 kinase | | | 2895 | |
| ATP1 | 0024 | | SKCa channel | | 0167 | | | Fyn kinase | | | 0212 | |
| BZD | 0028 | | Cl channel | | 0170 | | | HER/ErbB2 kinase | | | 1598 | |
| B2 | 0033 | | AMPA | | 0064 | | | IGF1R | | | 3061 | |
| CB1 | 0036 | | GABA Transporter | | 0060 | | | IRK | | | 2898 | |
| CCK1 | 0039 | | NMDA | | 0066 | | | PAK1 | | | 1934 | |
| ETA | 0054 | | Glycine | | 0068 | | | PKA | | | 2927 | |
| GABA | 0057 | | N α4β2 | | 3029 | | | PKCα | | | 0348 | |
| CXCR2 | 0419 | | N α7 | | 3010 | | | PKCβ1 | | | 2888 | |
| GAL2 | 0410 | | PCP | |  | | | PKCβ2 | | | 2750 | |
| CCR1 | 0361 | | NMDA | | 0066 | | | PKCγ | | | 0350 | |
| H1 | 0870 | | Kainate | | 0065 | | | RAF-1 kinase | | | 2936 | |
| H2 | 1208 | | AMPKinaseα | | 1572 | | | Src kinase | | | 2907 | |
| MC4 | 0420 | | CAMK1α | | 2739 | | | mTOR kinase | | | 2941 | |
| MT1 | 1538 | | CAMK2α | | 3024 | | | TRKA | | | 2901 | |
| NK2 | 0102 | |  | |  | | |  | | |  | |
| **Activity EC50 > 10,000 nM** | | | | | | | | | | | | |
| **Target** | **Catalog No.** | | **Target** | | | **Catalog No.** | | | **Target** | | | **Catalog No.** |
| alpha 1A (agonist) | 1500 | | 5HT2A (agonist) | | | 1023 | | | Beta 3 (antagonist) | | | 2191 |
| alpha 1B (agonist) | 1901 | | 5HT2B (agonist) | | | 1377 | | | D1 (antagonist) | | | 1686 |
| alpha 2A agonist) | 2558 | | 5HT2C (agonist) | | | 1221 | | | D3 (antagonist) | | | 684 |
| alpha 2B (agonist) | 1813 | | 5HT4e (agonist) | | | 1044 | | | D2S (antagonist) | | | 2569 |
| alpha 2C (agonist) | 1736 | | 5HT6 (agonist) | | | 1627 | | | D4.4 (antagonist) | | | 684 |
| Beta1 (agonist) | 1605 | | 5HT7 (agonist) | | | 1661 | | | 5HT1 A (antagonist) | | | 2101 |
| Beta 2 (agonist) | 1976 | | alpha 1A (antagonist) | | | 1501 | | | 5HT1B (antagonist) | | | 2604 |
| Beta 3 (agonist) | 2189 | | alpha 1B (antagonist) | | | 1902 | | | 5HT2A (antagonist) | | | 1024 |
| D1 (agonist) | 1685 | | alpha 2A (antagonist) | | | 2559 | | | 5HT2B (antagonist) | | | 1812 |
| D2S (agonist) | 2566 | | alpha 2B (antagonist) | | | 1814 | | | 5HT4e (antagonist) | | | 1045 |
| D4.4 (agonist) | 1699 | | alpha 2C (antagonist) | | | 1737 | | | 5HT6 (antagonist) | | | 1628 |
| 5HT1A (agonist) | 2093 | | Beta1 (antagonist) | | | 1606 | | | 5HT7 (antagonist) | | | 1662 |
| 5HT1B (agonist) | 2600 | | Beta 2 (antagonist) | | | 1977 | | |  | | |  |
