# Supplementary material for: Alzheimer's Therapeutics Targeting Amyloid Beta 1–42 Oligomers II: Sigma-2/PGRMC1 Receptors Mediate Abeta 42 Oligomer Binding and Synaptotoxicity
Source: PLoS One. 2014 Nov 12;9(11):e111899. doi: 10.1371/journal.pone.0111899 (PMC4229119; doi:10.1371/journal.pone.0111899)
Supplement: Table S2 — Genetic analysis of MAPR family members PGRMC1, PGRMC2, neudesin (NENF) and neuferricin. (DOCX) [file pone.0111899.s006.docx]

Supplemental Table S2

| **Gene** | **Chromosome** | **Single Nucleotide Polymorphism** | **Base Position** | **rsID** | **Alleles** | **Amino Acid change** | **AD (%MAF)** | **Controls (% MAF)** | **OR** | **P value** |
| --- | --- | --- | --- | --- | --- | --- | --- | --- | --- | --- |
| **NENF** | 1 | N/A | 212606392 | none | G/A | R36H | 0.06% | 0.00% | NA | 0.501 |
| **NENF** | 1 | exm147165 | 212617692 | none | C/T | R84X | 0.00% | 0.00% | NA | NA |
| **NENF** | 1 | exm2273742 | 212617723 | none | C/T | K94R | 0.00% | 0.00% | NA | NA |
| **NENF** | 1 | exm147172 | 212617740 | rs78790038 | A/G | I115M | 0.19% | 0.03% | 6.3 | 0.4817 |
| **NENF** | 1 | exm147177 | 212619173 | none | A/G | T114M | 0.00% | 0.08% | 0 | 0.21 |
| **NENF** | 1 | exm147180 | 212619182 | rs34254916 | T/C | M118T | 0.00% | 0.00% | NA | NA |
| **NENF** | 1 | exm147185 | 212619247 | none | C/T | V140I | 0.00% | 0.00% | NA | NA |
| **NENF** | 1 | exm2250675 | 212619339 | rs4804 | C/T | D170D | 41.40% | 40.10% | 1.059 | 0.4291 |
| **PGRMC1** | X | exm2203765 | 118370441 | none | A/G | intergenic | 0.00% | 0.00% | NA | NA |
| **PGRMC1** | X | exm1654922 | 118370489 | rs145582672 | A/G | S55G | 0.00% | 0.00% | NA | NA |
| **PGRMC1** | X | exm1654930 | 118377123 | rs147630867 | G/A | R165H | 0.24% | 0.24% | 1.096 | 0.7644 |
| **PGRMC1** | X | N/A | 118377148 | none | G/C | E173D | 0.00% | 0.03% | 0 | 0.654 |
| **PGRMC2** | 4 | exm424472 | 129208629 | rs149247614 | C/T | E130G | 0.00% | 0.00% | NA | NA |
| **PGRMC2** | 4 | exm424475 | 129208660 | none | A/G | P120S | 0.00% | 0.08% | 0 | 0.95 |
| **PGRMC2** | 4 | N/A | 129208728 | none | A/G | V97A | 0.00% | 0.08% | 0 | 0.75 |
| **PGRMC2** | 4 | N/A | 129208957 | none | A/C | W21G | 0.06% | 0.08% | 0.85 | 0.68 |
| **PGRMC2** | 4 | N/A | 129209001 | none | C/A | R6L | 0.00% | 0.17% | 0 | 0.85 |
| **Neuferricin** | 17 | exm1279848 | 4047062 | rs146655031 | A/G | R5G | 0.13% | 0.08% | 1.5 | 0.5708 |
| **Neuferricin** | 17 | exm1279859 | 4047227 | rs140071359 | T/C | F60L | 0.13% | 0.08% | 1.5 | 0.5708 |
| **Neuferricin** | 17 | N/A | 4053214 | none | G/A | G94R | 0.03% | 0.00% | NA | 0.97 |
| **Neuferricin** | 17 | exm1279861 | 4047255 | rs150241046 | A/G | D69G | 0.00% | 0.00% | NA | NA |
| **Neuferricin** | 17 | exm1279879 | 4058001 | rs144648688 | T/G | V142G | 0.00% | 0.13% | 0 | NA |
| **Neuferricin** | 17 | exm1279885 | 4058024 | rs142632874 | T/C | Q150X | 0.00% | 0.00% | NA | 0.2102 |
| **Neuferricin** | 17 | exm1279890 | 4058094 | rs144989131 | T/C | L173P | 0.00% | 0.00% | NA | NA |
| **Neuferricin** | 17 | exm1886987 | 4058111 | rs142095753 | C/T | W179A | 0.00% | 0.00% | NA | NA |
| **Neuferricin** | 17 | exm1886988 | 4058153 | rs151170515 | C/T | S193G | 0.00% | 0.00% | NA | NA |
| **Neuferricin** | 17 | exm1279897 | 4060227 | rs149212394 | T/C | C216R | 0.00% | 0.00% | NA | NA |
| **Neuferricin** | 17 | exm1279898 | 4060233 | rs118123280 | A/G | M218V | 1.66% | 1.10% | 1.49 | 0.25 |
| **Neuferricin** | 17 | exm1279905 | 4060345 | none | T/C | L255P | 0 | 0 | NA | 0.1683 |

**Supplemental Table 2.** **Genetic analysis of MAPR family members PGRMC1, PGRMC2, neudesin (NENF) and Neuferricin**. Coding sequence variants in these genes were extracted from the Illumina exome-chip (Infinium HumanExome BeadChip Kit) from 1,032 Alzheimer’s disease cases and 657 nondemented elderly controls, and from the ADNI whole-genome sequencing data (200 controls and 200 AD cases). The Illumina Exome-array contains > 250,000 exonic polymorphisms, many resulting in coding changes to the protein, representing diverse populations—including European, African, Chinese, and Hispanic individuals—and a range of common conditions, such as type 2 diabetes, cancer, metabolic, and psychiatric disorders. These genes (NENF, PGRMC1 and 2) are also highly conserved among mammals (>95% amino acid identity between humans, chimps, macaque, cow, dog, mouse and rat; see Supplemental Figure 10). Together, these data indicate that there is little coding variability in these genes (lower than expected by chance) suggesting that they are essential genes. AD (%MAF)= minor allele frequency in Alzheimer’s disease cases (%), Controls (%MAF) = minor allele frequency in Controls (%)
